# Structural insights into the mosaic and modular organization of fourteen co-occurring resistance genes in multidrug resistant *Enterobacteriaceae*

**DOI:** 10.1371/journal.pone.0355393

**Published:** 2026-08-26

**Authors:** Connor A. J. Rossel, Lieke B. van Alphen, Edou R. Heddema, Paul H. M. Savelkoul

**Affiliations:** 1 Department of Medical Microbiology, Zuyderland Medical Center, Sittard-Geleen, The Netherlands; 2 Department of Medical Microbiology, Infectious diseases and Infection prevention, Care and Public Health Research Institute (CAPHRI), Maastricht University Medical Center, Maastricht, The Netherlands; University of Cape Town, SOUTH AFRICA

## Abstract

Up to 14 co-occurring antimicrobial resistance genes (ARG) were previously detected in multiple outbreaks in the Netherlands involving various *Enterobacteriaceae* species, clones and plasmids, but similar multidrug resistance regions (MRR). This study aimed to map the reported occurrence, mosaic and modular genetic context of the involved ARG. Google Scholar and PubMed Central were queried for articles describing long-read assemblies with ≥ 6 co-occurring ARG. Plasmid (*n* = 119) and chromosomal (*n* = 8) sequences of 9 bacterial taxa originating from North America (*n* = 39), Europe (*n* = 34), Africa (*n* = 16), Asia (*n* = 14), Oceania (*n* = 13) and South America (*n* = 7) were extracted from NCBI. Based on colocalized insertions sequences, transposons and integrons, the ARGs could be divided into nine distinct resistance elements. These elements presented a total of 118 mosaic structural variants (SV): *qnrB1* (SV = 27; *n* = 102), *bla*_CTX‑M‑15_ (SV = 23; *n* = 137), *tet*(A) (SV = 18; *n* = 93), *dfrA14* (SV = 14; *n* = 115), *sul2—strA—strB* (SV = 13; *n* = 120), *bla*_TEM‑1b_ (SV = 12; *n* = 119), *aac(6’)‑Ib‑cr*—*bla*_OXA‑1_—*catB3* (SV = 7; *n* = 137), *aac(3)‑IIe* (SV = 2; *n* = 92), and *aadA1*—*catA1* SV = 2; *n* = 12). The MRR showed high modularity with no clear ARG order. Still, three frequent sub-configurations were found. Firstly, *bla*_TEM‑1b_ was flanked by *sul2—strA—strB* downstream (92.6%) and *bla*_CTX‑M‑15_ upstream (84.0%). Secondly, *qnrB1* was flanked by *tet*(A) upstream (62.4%) and *dfrA14* (63.4%) downstream. Thirdly, *aac(3)‑IIe* was flanked by *aac(6’)‑Ib‑cr*—*bla*_OXA‑1_—*catB3* upstream (80.4%). In conclusion, the 14 ARG co-occur globally in similar MRR that are complex to interpret due to modularity and mosaicism. Still, the preservation of these clinically relevant resistance elements infers a potential means of transfer between distinct *Enterobacteriaceae* isolates.

## Introduction

Antimicrobial resistance (AMR) in bacterial pathogens is a major global burden that is associated with and attributable to patient mortality [1]. This challenge is compounded by the increasing co-occurrence of antimicrobial resistance genes (ARG), which has led to a rise in difficult-to-treat multidrug resistant (MDR) bacteria. To manage infections caused by these bacteria, there has been an urgent need for the development of novel therapeutics [2]. In addition, it has become evident that the role of ARG characterization and tracing is essential to limit the rising dissemination of AMR in the clinical setting [3]. However, effective infection prevention control measures are frequently challenged by the extensive variety and complexity of ARG dissemination, which complicates epidemiological investigations that are commonly based on clonal transmission analysis. Multiple nested genetic levels must be accounted for, e.g., clones and mobile genetic elements (MGE) such as plasmids [4].

Other MGE such as insertion sequences (IS), transposons (Tn) and integrons (In) provide intracellular mobility of ARG through transposition, homologous- and site-specific recombination [5,6]. These mechanisms may give rise to novel variants of a sequence structure (i.e., mosaicism) due to insertion of additional genetic content and deletion or rearrangement of pre-existing regions. For instance, characterization of the *bla*_NDM_ genes and its immediate genetic context showed multiple highly conserved structural variants that differed upstream of *bla*_NDM_ through the association with various MGE such as IS*Aba125*, IS*26* and IS of the IS*5* family [7]. These mechanisms commonly aggregate MGE and ARG into multidrug resistance regions (MRR), conferring MDR phenotypes [5]. The integration of MRR in a wide variety of plasmid types has allowed for mobility between bacterial species and strains by means of conjugation [6,8,9].

Within the Netherlands, several outbreaks of MDR *Enterobacteriaceae* have been documented, with similar isolate resistomes harboring as many as 14 co-occurring ARG [10–12]. These genes confer resistance to first- and second-line antimicrobials: to aminoglycosides by *aac(3)‑IIe*, *aac(6’)‑Ib‑cr*, *aadA1*, *strA* and *strB*; to extended spectrum β‑lactams by *bla*_CTX‑M‑15_; to broad spectrum β‑lactams by *bla*_TEM‑1b_ and *bla*_OXA‑1_; to fluoroquinolones by *qnrB1* and *aac(6’)‑Ib‑cr*; to chloramphenicol by *catA*1 and *catB3*; to trimethoprim by *dfrA14*; to sulfonamides by *sul2*; and to tetracyclines by *tet*(A). It was striking that these co-occurring ARG were associated with different *Enterobacteriaceae* species, clones, and plasmids. While these ARG appeared co-localized in similar but structurally unidentical MRR, an extensive analysis was unattainable since only a few isolates were long-read sequenced [12]. Therefore, questions remained with respect to occurrence and structure of these co-occurring ARG in MRR. Other studies have similarly indicated that these ARG are co-localized in MRR of various *Enterobacteriaceae* taxa in different contexts: Bloodstream infections with *Klebsiella pneumoniae* in the USA [13]; Veterinarian infections with *Escherichia coli* in Italy [14]; And dissemination of *Enterobacter* spp. within a one-health setting in Guadeloupe [15].

To expand on this observation, we first aimed to obtain an overview of studies which have reported on the co-occurrence of these ARGs through long-read sequencing. This provided a diverse dataset which consisted of sequences originating from various genomic contexts (i.e., distinct species, clones, and plasmids) and geographically distinct areas. Secondly, recombination events within individual resistance elements (i.e., an ARG associated with specific IS, Tn or In) may result in mosaic variation of the structure [5]. We aimed to identify the mosaic structural variants of resistance elements associated with the 14 co-occurring ARG to understand whether certain variants are more common than others. Thirdly, MRR are modular structures in which the individual resistance elements can be arranged in various configurations. However, the number of distinct configurations is lower than would be expected of an *ad random* assembly [16]. Therefore, we aimed to analyze regular configurations between these 14 ARG by mapping the flanking regions of each resistance elements.

## Methods

### Literature assessment

A structured literature review with an ARG-centric search method was used to explore the literature for genomic sequences containing the 14 ARG in question, for which search criteria were drafted. Firstly, both Google Scholar and PubMed Central were queried with a range from January 2014 to March 2024. Secondly, since querying all 14 ARG would retrieve few to no results, the choice was made to query multiple combinations of three to five ARG of interest (S1 Table). Thirdly, we aimed to analyze the structure of the resistance elements, for which long-read sequencing data was required. Therefore, “Nanopore”, “Pacific Biosciences” and “Whole Genome Sequencing” were added to the query. Lastly, the main body and supplementary materials of the articles were screened, and a genomic sequence was considered of interest when it incorporated at least six (≥6) associated ARG. The rationale to search for 6–14 co-occurring ARGs is based on observations at our medical centers in the Netherlands [12]. Here we noticed that as little as six of these ARGs could already infer MDR phenotypes against (global) cornerstone antimicrobial categories for the treatment of *Enterobacteriaceae* infections. This includes non-susceptibility to aminoglycosides by *aac(3)‑IIe* or *aac(6’)‑Ib‑cr*, to cotrimoxazole by *dfrA14* and *sul2*, to fluoroquinolones by *qnrB1* or *aac(6’)‑Ib‑cr*, and to extended-spectrum β‑lactams by *bla*_CTX‑M‑15_. This approach allowed for possible genomic plasticity such as potential deletions, insertions, and structural rearrangements. Articles which fulfilled these criteria were further studied. This included the examination of the articles for the NCBI nucleotide accession numbers of the genomic sequences and the associated metadata. Additional selection based on assembly quality thresholds was not applied. The available FASTA assemblies were extracted from GenBank for comparative analysis.

### Sequence analysis and annotation

The presence of ARGs in the chromosomal and plasmid sequences was confirmed with AMRFinderPlus v3.12.8 [17]. The obtained resistance markers were plotted into a heatmap in Excel. The sequences were reannotated with Bakta v1.9.2 [18] to attain a uniform baseline annotation, which was subsequently uploaded in Geneious Prime V2023.1.1 [19]. The sequences were further annotated for (truncated) ARG in Geneious Prime with BLAST (≥25% minimum coverage) using the Bacterial Antimicrobial Resistance Reference Gene Database (PRJNA313047) dataset to remain congruent with the AMRFinderPlus reference dataset [17]. Integrons were annotated with IntegronFinder v2.0.3 [20]. The IS and Tn including the left terminal inverted repeat (IRL) and right terminal inverted repeat (IRR) were annotated with BLAST with the help of the ISFinder [21] and TnCentral [22] databases. Discrepancies between annotation tools were resolved by manual assessment of the locus in Geneious Prime. The annotations of IS, Tn or Integrons by their respective tools took precedence over Bakta annotations. This is especially important for regions rich in IS- and Tn-elements, since Bakta only infers transposase or resolvase CDS but does not annotate the inverted repeats which are necessary to mark the flanks of these elements.

### Characterization of resistance elements

The resistance elements which incorporated the 14 ARG were first defined under the assumption that modular rearrangements introduce different configurations in which the ARG are organized on the sequences. Accordingly, it was first outlined if certain ARG were organized next to each other throughout the dataset. The ARG that were consistently organized together (e.g., through tandem arrangement) were grouped into a single resistance element, otherwise an ARG was assigned to its own element. Per resistance element, the flanking genetic context was then assessed upstream and downstream of the ARG. This was done in an iterative manner—considering the modular rearrangements—to distinguish IS, Tn, integrons or other genes specific to certain resistance elements. This allowed for the demarcation of resistance element boundaries, which were typically marked by an IS or Tn. However, some considerations were made with the demarcation of these boundaries. First, the same IS*26* could be assigned to two distinct resistance elements within a single sequence. This takes into account IS*26*-mediated rearrangements between resistance elements through targeted conservative transposition and the potential formation of a pseudo-compound transposon (array) [23]. Secondly, in some cases no clear boundary could initially be demarcated between two frequently flanking resistance elements. This was the case for Tn*5403* separating the *tet*(A) and *qnrB1* resistance elements, and for IS*Ecp1* separating the *bla*_CTX‑M‑15_ and *bla*_TEM‑1_ resistance elements. Upon inspection of sequences where these resistance elements did not flank each other, (partial) Tn*5403* and IS*Ecp1* annotations were detected at the boundaries of both their respective resistance elements and thus assigned as a boundary for both. Lastly, some mosaic variants of resistance elements were demarcated with a truncated IS or Tn. These truncated boundaries were assigned due to apparent insertion of another IS or Tn that has already been assigned to a different resistance element. Alternatively, truncated boundaries could also be assigned based on an apparent deleterious event without evident involvement of an IS or Tn. In cases where no clear boundary could be indicated by an IS or Tn, these were drawn comparatively based on sequence similarity and coverage with the predominant mosaic variant as reference using BLAST in Geneious Prime. The terminal inverted repeats were also assessed as these might play a role in the potential mobility of the resistance element. The mosaic structural variants are named “v” (from variant) followed by the length of the respective sequence. The resistance elements were visualized with Geneious Prime and Inkscape. The flanking regions were also counted in Geneious Prime and visualized with ggplot2 v3.5.1 [24] and ggalluvial v0.12.5 [25].

### Ethics statement

Ethical approval was not required for this study.

## Results

### Literature screening

**Fig 1:**
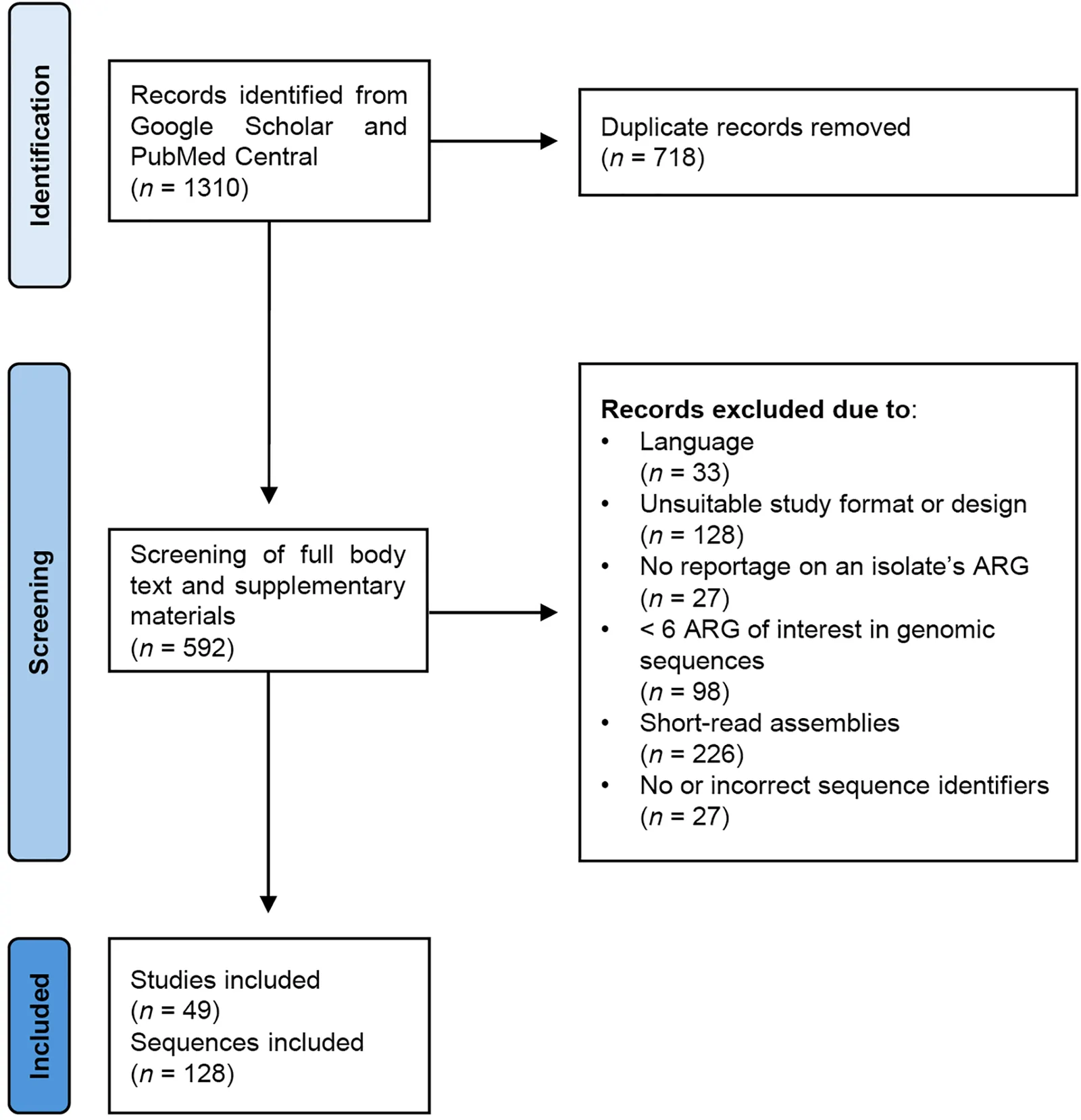
Flow diagram of the structured literature review process.

The identification, screening and selection process is outlined in Fig 1. The database search generated a total of 1310 hits derived from 12 query inputs. After deduplication, only 592 hits remained. These articles were screened for the criterion of at least 6 ARG associated in a single genomic sequence, which resulted in a total of 49 articles published between 2014 and 2024, 124 bacterial isolates, and 128 genomic sequences (S2 Table) [13–15, 26–71]. The main reasons for the exclusion of articles were publication in a language other than English (*n* = 33); unsuitable study format or design (e.g., narrative review, congress abstract, dissertation, preprint, meta-analysis, use of metagenome sequencing or absence of whole genome sequencing)(*n* = 132); absence of reportage on an isolate’s ARG carriage (*n* = 27); less than 6 ARG of interest in a single genomic sequence or isolate (*n* = 98); and absence of long-read assemblies (*n* = 226) or correct sequence identifiers (*n* = 27). The last search was updated on 2nd April 2024.

### Overview of the co-occurring ARG in the literature

The 123 MDR bacterial isolates originated from varying geographic regions with global representation between 2009 and 2022, which includes North America (*n* = 39), Europe (*n* = 34), Africa (*n* = 16), Asia (*n* = 14), Oceania (*n* = 13), and South America (*n* = 7)(see also S2 Table for a characteristics overview of included sequences). Most isolates were derived from human hosts (*n* = 110), with the remainder being from animals (*n* = 9) or the environment (*n* = 4). The isolate species mainly belonged to the *Enterobacteriaceae* family (121/123). The most predominant taxa were *Klebsiella pneumoniae* complex (*n* = 92), followed by *Enterobacter cloacae* complex (*n* = 14), *Escherichia coli* (*n* = 9), *Klebsiella oxytoca* complex (*n* = 2), *Citrobacter freundii* complex (*n* = 2), *Klebsiella aerogenes* (*n* = 1), *Salmonella enterica* (*n* = 1), *Serratia marcescens* (*n* = 1) and *Shewanella* sp. (*n* = 1). The co-occurring ARG appeared primarily located on plasmids (*n* = 119), and infrequently on chromosomes (*n* = 8). Three isolates presented the ARG simultaneously on the chromosome and the plasmid, while a single isolate contained multiple distinct plasmids (S2 Table).

**Fig 2:**
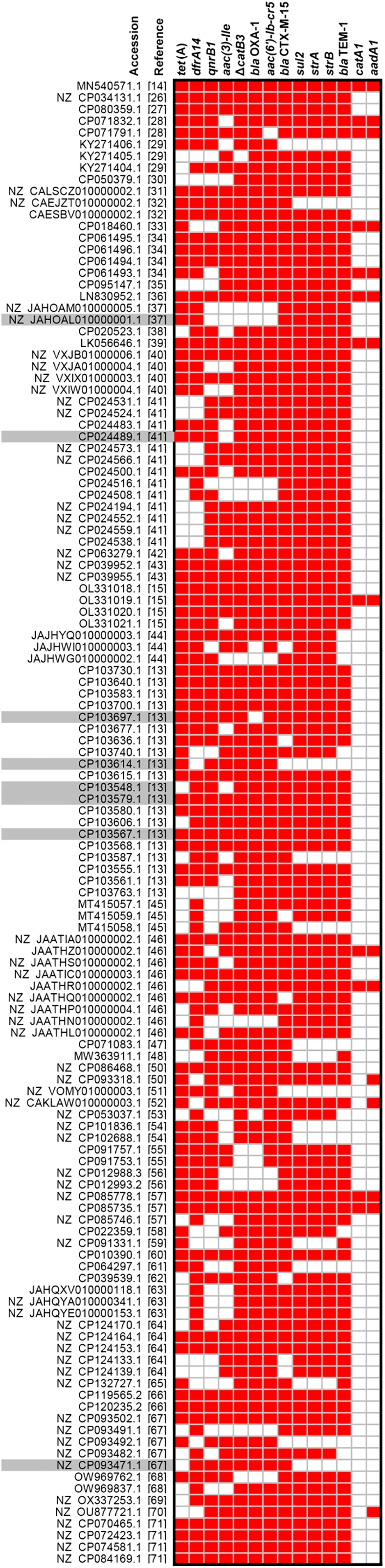
Heatmap of the 14 ARG found in the literature sequences. Presence of the ARG is indicated in red filled squares. Accessions of chromosomal sequences are shaded gray, plasmid sequences remain unshaded.

Overall, a mean of 10.2 associated ARGs was identified per sequence (Fig 2). The *bla*_CTX‑M‑15_ and *aac(6’)‑Ib‑cr* ARG appeared to be present in the most sequences (*n* = 116), followed by *bla*_OXA‑1_ (*n* = 112), *catB3* (*n* = 114), *strA* (*n* = 112), *strB* (*n* = 112), *sul2* (*n* = 112), *bla*_TEM‑1_ (*n* = 108), *dfrA14* (*n* = 101), *qnrB1* (*n* = 93), *aac(3)‑IIe* (*n* = 86), *tet*(A) (*n* = 85), *aadA1* (*n* = 15), *catA1* (*n* = 12). The *catB3* was truncated (≤70% coverage) in all instances, which does not confer resistance to chloramphenicol [72]. It should be noted that 27 of 127 sequences carried up to five copies of one ARG (S3 Table). Resistance to carbapenems was further evaluated, since it could provide a last line therapeutical alternative to pathogens which carry the ARG of interest. Almost half of the isolates (*n* = 58) carried a carbapenemase gene (S3 Table), which was infrequently incorporated into the plasmid and chromosome sequences of interest (*n* = 9)(S1 Fig). Similarly, there was a sparse number of plasmids that incorporated other ARGs (32 of119 plasmids), with a median of 1.5 other ARGs in these plasmids.

### Mosaicism of the resistance elements

To define the mosaicism associated with the various resistance elements, the direct genetic context was analyzed of each ARG or multiple adjoined ARG. Overall, 9 distinct resistance elements could be distinguished, each presenting several mosaic structural variants, for a total of 118 mosaic structural variants (Table 1 and S2 Fig). At the minimum, four resistance elements were found in sequences containing 6 ARG of interest.

**Table 1:** Mosaic structural variants of resistance elements and their observed frequency.

Element 1: *aac(6’)‑Ib‑cr*—*bla*_OXA‑1_—Δ*catB3*		Element 2: *aac(3)‑IIe*		Element 3: *aadA1—catA1*		Element 4: *qnrB1*		Element 5: *tet*(A)		Element 6: *dfrA14*		Element 7: *bla*_CTX‑M‑15_		Element 8: *bla*_TEM‑1b_		Element 9: *Sul2—strA—strB*	
Variant	*n* =	Variant	*n* =	Variant	*n* =	Variant	*n* =	Variant	*n* =	Variant	*n* =	Variant	*n* =	Variant	*n* =	Variant	*n* =
v3826	129	v4240	91	v5317	9	v8323	57	v10461	56	v8340	77	v6037	60	v3781	104	v7085	72
v3466	2	v2668	1	v5792	3	V9253	8	v5358	17	v9167	16	v6594	43	v2247	3	v7383	19
v2769	2					v8250	7	v4398	3	v2791-7304	8	v5915	4	v2822	3	v6016	9
v3224	1					v7510	5	v7523	2	v3920	3	v5929	4	V4477	3	v4786	6
v3583	1					v10150	2	v8646	2	v6428	2	v5890	3	v2043	2	v3817	3
v3634	1					v4657	2	v10019	1	v3458	1	v5908	3	V4043	2	v1921-6811	2
v4654	1					v11354	1	v10372	1	v3919	1	v4943	2	v2341	2	v8327	2
						v11703	1	v11080	1	v6180	1	v5386	2	v2796	2	v2622	1
						v11716	1	v2930	1	v8115	1	v5801	2	v5182	2	v5290	1
						v12092	1	v4953	1	v8201	1	v2800	1	v1847	1	v6026	1
						v13043	1	v7157	1	v8633	1	v5052	1	V4424	1	v7217	1
						v13807	1	v7661	1	v8711	1	v5358	1	V4580	1	v7412	1
						v3433	1	v7786	1	v9319	1	v5376	1			v8003	1
						v5175	1	v7868	1	v9395	1	v5482	1				
						v5870	1	v8152	1			v5756	1				
						v6017	1	v8548	1			v5943	1				
						v6089	1	v9596	1			v6313	1				
						v6988	1	v9674	1			v6324	1				
						v7117	1					v6988	1				
						v7231	1					v7103	1				
						v7437	1					v7339	1				
						v8015	1					v7421	1				
						v8122	1					V7660	1				
						v8258	1										
						v8867	1										
						v9059	1										
						v9648	1										

### Resistance element 1: *aac(6’)-Ib‑cr– bla*_OXA‑1_*–* Δ*catB3*

**Fig 3:**
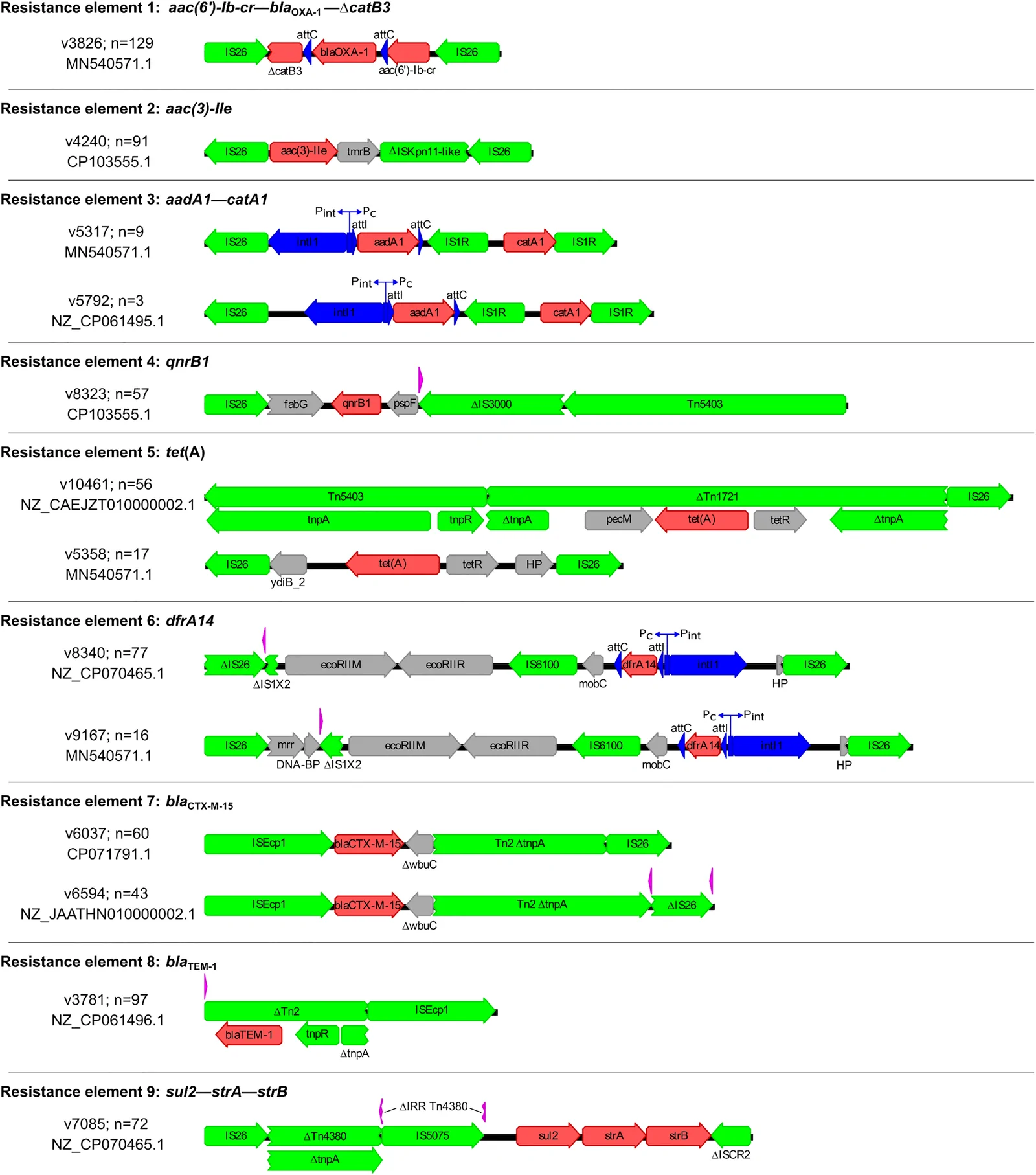
Genetic structures of the most frequent structural variants of the nine resistance elements. The red arrows indicate ARG. The green arrows indicate *IS*- and *Tn*-elements. The blue arrows indicate integron integrase, attI, attC and promotor sites. Arrows with a serrated end present annotations of truncated sequences. The purple arrows indicate complete terminal inverted repeats of a truncated *IS-* or *Tn*-elements.

The first resistance element contains *aac(6’)‑Ib‑cr*, *bla*_OXA‑1_ and Δ*catB3* which were arrayed in tandem. A total of 7 variants were found among 137 occurrences. In all variants, the ARG were included in partial gene cassettes with sequence similarity to In*37* [73,74]. These partial cassettes were always flanked by two IS*26* which likely mediated the *catB3* truncation and have been commonly described [72,74–77]. In the current dataset, the typical variant v3826 (129/137) is flanked by two inversely orientated IS*26* (Fig 3) and is therefore not a PCT [23].

### Resistance element 2: *aac(3)‑IIe*

The second resistance element includes *aac(3)‑IIe* which was only found in 2 variants among 92 occurrences (S2 Fig). Both variants were IS*26* PCT, of which one contained an IS*26* mediated deletion (Fig 3). This genetic context and the dominant variant v4240 (91/92) have previously been described [5,75].

### Resistance element 3: *aadA1 – catA1*

The third resistance element incorporates *aadA1* and *catA1*. The choice was made to analyze both ARG as one resistance element since they appeared simultaneously absent or present (Fig 3) and co-localized on the periphery of the multidrug resistance regions. This element appeared conserved with only 12 occurrences and 2 variants. These variants differed by 475 bp between IS*26* and a class 1 integron with an *aadA1* cassette (Fig 3). This integron appeared akin to elements of In*2* in the Tn*21* transposon [78]. Downstream of *aadA1* lies a Tn*9*‑like structure with an IS*1R* upstream of *catA1*, but with an inverted IS*1R* inserted 7‑bp into the downstream end of *catA1*. While both the In*2* derivative and the Tn*9*‑like structure suggest a resemblance to the Tn*21*‑like element Tn*2670* [78], this region appears to consist of a unique composition.

### Resistance element 4: *qnrB1*

This resistance element includes *qnrB1* and presents frequent incorporation of various flanking IS- and Tn-elements. In total, 27 variants were detected among 102 occurrences. The variants were characterized by an IS*26* downstream of *qnrB1* and an IS*3000* which was located upstream (22/27 variants; 96/102 occurrences). The IS*3000* appeared primarily truncated at the IRL side by insertion of Tn*5403* (79/102) (S2 Fig), as has been described previously [79,80]. Even though relatively many variants were detected in this resistance element, over half (57/102) of the occurrences were v10461 (Fig 3).

### Resistance element 5: *tet*(A)

This resistance element carries *tet*(A) and presents a total of 18 variants among 93 occurrences. These variants vary in size, between 2930 bp to 11080 bp. Still, 17/18 variants and 76/93 of these occurrences were characterized by the incorporation of *tet*(A) into partial Tn*1721* derivatives (S2 Fig) [5]. For example, the most common variant (56/93 occurrences) is flanked by an IS*26* at the IRL side of Tn*1721*, while the right IRR side was truncated by the insertion of Tn*5403* (Fig 3). A single variant (v5358) was not characterized as a derivative of Tn*1721* and is flanked by two inversely oriented IS*26* elements (17/93 occurrences) (Fig 3). Pairwise alignment showed an identity of 94.9% for *tet*(A) and 93.4% for *tetR*(A) between v5358 and the common Tn*1721* derivative v10461. This suggests an independent capture of *tet*(A) and *tetR*(A) from a different source organism, potentially by IS*26*. Altogether, 13 of these 17 v5358 occurrences were derived from *Enterobacter* sp. isolates.

### Resistance element 6: *dfrA14*

This resistance element contains *dfrA14* and presents 14 variants among 115 occurrences. It appeared as the cassette of a class 1 integron, which was in turn nested between IS elements (113/115 occurrences)(S2 Fig). These elements were usually an IS*26* located upstream and an IS*6100* located downstream of *dfrA14* (109/115 occurrences). Furthermore, the restriction-modification system EcoRII was located adjacent to the IS*6100* and is in turn flanked by an IS*26*, which was directly oriented with the IS*26* upstream of *dfrA14*. Therefore, these common variants (e.g., V8340 and V9167 in Fig 3) can be described as a PCT. Lastly, it should be mentioned that the two variants v2791‑V7304 (8 occurrences) and v6428 (*n* = 2) carried a truncated *dfrA14* (<60% coverage), which likely do not confer the associated trimethoprim phenotype anymore.

### Resistance element 7: *bla*_CTX‑M‑15_

This resistance element includes *bla*_CTX‑M‑15_ and presents 23 variants among 137 occurrences. In accordance with the literature, the *bla*_CTX‑M‑15_ was part of the 2971 bp transposition unit (TPU) IS*Ecp1*–*bla*_CTX‑M‑15_–Δ*wbuC* (S2 Fig) [81], which appeared complete in 110/137 occurrences. This TPU was also typically inserted into Tn*2* (136/137), as has been reported [81]. The Tn*2* IRR was frequently flanked by IS*26* and here two major variants could be distinguished (Fig 3). In the first variant (v6037, 60/137 occurrences), the IRR end of Tn*2* is truncated by IS*26* which leads to a 574 bp deletion. In the second variant (v6594, 43/137 occurrences), the IRR end of Tn*2* is complete and is flanked by IS*26* with a truncated IRL due to a 27 bp deletion.

### Resistance element 8: *bla*_TEM‑1b_

This resistance element includes *bla*_TEM‑1b_ and presents 12 variants among 119 occurrences. It is part of an incomplete Tn*2* in all occurrences, mainly due to insertion of IS*Ecp1*–*bla*_CTX‑M‑15_–Δ*wbuC* TPU (S2 Fig). We chose to present the common variant v3781 (97/119) that has been interrupted by the TPU with a flanking IS*Ecp1* (Fig 3) in order to differentiate it from the other variants in which ΔTn*2* has been (further) interrupted by IS*26* (S2 Fig).

### Resistance element 9: *sul2* —*strA* — *strB*

This resistance element includes *sul2*, *strA* and *strB* which were positioned in tandem and in direct orientation with each other. A total of 120 occurrences were registered alongside 13 variants (S2 Fig). The three ARG were downstream frequently flanked by an incomplete IS*CR2* (115/120 occurrences), a structure which is partially similar as described in the RSF1010 IncQ plasmid [5]. It has been suggested that this structure was created through transposition of Tn*5393* (which includes *strA* and *strB*) into the IS*CR2*‑*sul2* segment, with an additional deletion event [5,82]. However, the ARG in our variants appeared commonly flanked by a partial Tn*4380* (Tn*21*‑like) [83] with an interrupted IRR by IS*5075* insertion (114/120 occurrences) [84], and a truncated IRL side through insertion of IS*26* (114/120 occurrences)(Fig 3). Lastly, it should be mentioned that the minor variant v1921‑v6811 (2 occurrences) carried a truncated Δ*strB* which likely does not confer the associated resistance phenotype anymore.

### Sensitivity analysis

The initial threshold for selecting sequences was based on prior observations in the Netherlands where at least 6 co-occurring ARG could be linked to a similar MDR phenotype. Because applying this threshold might have inadvertently skewed the main findings in the current global structural analysis, a post-hoc analysis was used to incrementally assess the impact of different ARG thresholds (from ≥6 to ≥14 ARG) on the retention of unique structural mosaic variants (S4 Table). The ratio of genomic sequences to unique mosaic variants appeared proportionate between the thresholds ≥6 and ≥12 ARG (ratio of 0.94 to 1.22). Increasing the threshold does not appear to heavily impact the frequently observed (*n* > 10) mosaic variants of each resistance elements. For instance, should a threshold of ≥8 ARG have been chosen, these frequently observed variants would have decreased by 18.6% at the most (from 43 to 35 for *bla*_CTX‑M‑15_ v6594). This appears proportionate to a decrease of genomic sequences from 127 to 105 (17.3%) or a decrease of unique low-frequency mosaic variants (*n* < 10) from 118 to 99 (16.1%).

### Modularity of the resistance elements

**Fig 4:**
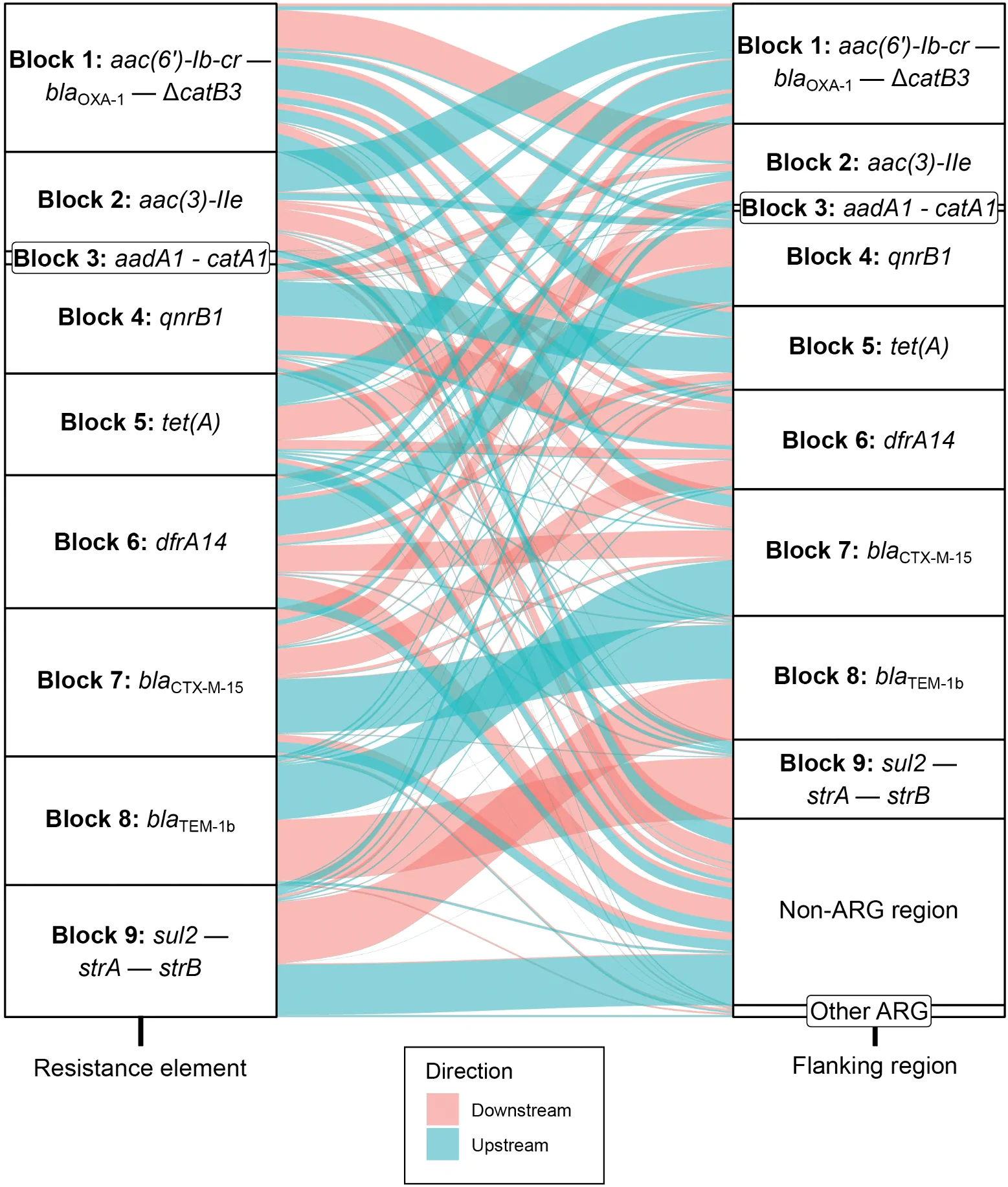
Alluvial plot of the resistance elements (left) and their flanking regions (right). The color of the line indicates whether a flanking region is found downstream (red) or upstream (blue) of a resistance element. Increased thickness of the line indicates a higher frequency in which a flanking region is found upstream or downstream of a resistance element.

Next, the MRR modularity was analyzed. While MGE associated transposition and recombination events may introduce rearrangements, deletions or insertions, certain resistance elements may be more frequently colocalized. To assess the ARG orientation and organization, the adjacent regions located directly upstream and downstream of the ARG were mapped, regardless of the mosaic variant (Fig 4 and S5 Table). The *aadA1*–*catA1* element was always found on the periphery of MRRs and flanked a non-ARG region (downstream; 12/12 occurrences), while it also flanked *sul2*—*strA*—*strB* (upstream; 11/12 occurrences). Similarly, the *sul2*—*strA*—*strB* was often located on the periphery as indicated by the number of non-ARG regions upstream (76.2%; 93/122 occurrences). However, it was also frequently flanked by *bla*_TEM‑1b_ elements downstream (92.6%; 113/122 occurrences). In turn, *bla*_TEM‑1b_ was frequently flanked upstream by *bla*_CTX‑M‑15_ (84.0%; 100/119 occurrences). The sequential co-occurrence and orientation of the *sul2*—*strA*—*strB, bla*_TEM‑1b_ and *bla*_CTX‑M‑15_ (*n* = 96) provides a large PCT of *circa* 15 kbp due to two directly oriented IS*26* at the extremities of this element. Downstream, *bla*_CTX‑M‑15_ was flanked by three resistance elements which made up 78.8% of the cases, these were *dfrA14* (48/137 occurrences), *aac(3)‑IIe* (37/137 occurrences) and *aac(6’)‑Ib‑cr*—*bla*_OXA‑1_—Δ*catB3* (23/137 occurrences).

The qnrB1 element was mostly flanked upstream by *tet*(A) (62.4%; 63/101 occurrences) and flanked downstream by *dfrA14* (63.4%; 64/101 occurrences). The sequential co-occurrence and orientation of the *dfrA14*, *qnrB1* and *tet*(A) (*n* = 41) could also provide a large PCT of *circa* 22 kbp.

The *aac(3)‑IIe* element was flanked downstream by three regions which made up 89.1% of the occurrences, these were *bla*_CTX‑M‑15_ (37/92 occurrences), non-ARG regions (32/92 occurrences) and *dfrA14* (13/92 occurrences). In contrast, *aac(3)‑IIe* was mostly flanked upstream by *aac(6’)‑Ib‑cr*—*bla*_OXA‑1_—Δ*catB3* (80.4%; 74/92 occurrences). In turn, *aac(6’)‑Ib‑cr*—*bla*_OXA‑1_—Δ*catB3* was flanked upstream by five regions which made up 89.1% of the occurrences, these include *tet*(A) (45/138 occurrences), non-ARG regions (32/138 occurrences), *bla*_CTX‑M‑15_ (22/138 occurrences), *dfrA14* (12/138 occurrences) and *qnrB1* (12/138 occurrences). While the *aac(3)‑IIe* and *aac(6’)‑Ib‑cr*—*bla*_OXA‑1_—Δ*catB3* are frequently co-localized, they cannot be defined as PCT due to inversely oriented IS*26* at their peripheries. It is therefore likely that these elements are part of larger mobile segments.

## Discussion

This study analyzed the genetic context associated with the co-occurrence of up to 14 ARG that were previously linked to various *Enterobacteriaceae* outbreaks in multiple Dutch hospitals [10–12]. Firstly, we aimed to obtain an overview of the genomic context in which these co-occurring ARG were reported. Therefore, genomic long-read sequences published in peer-reviewed articles were compiled through an ARG-centric query approach, which allowed for the collection of well-curated sequences with complete metadata. This provided an overview of the co-occurring ARG that iterated the global dissemination on plasmid sequences of various *Enterobacteriaceae* species in clinical settings. Additionally, the ARG of interest appeared only sporadically together with other ARG on the same plasmid, although they were frequently reported in carbapenemase-producing *Enterobacteriaceae* (CPE) isolates.

Secondly, we aimed to analyze the mosaic resistance elements that were associated with the 14 co-occurring ARG, and whether dominant variants could be detected. To limit the potential influence of variation arising from modularity (i.e., rearrangement between resistance elements), it was chosen to analyze only the direct environment of each ARG. Overall, 9 distinct resistance elements could be distinguished, each presenting several mosaic variants. Some resistance elements were well preserved with only a few variants (e.g., *aac(6’)‑Ib‑cr*—*bla*_OXA‑1_—Δ*catB3* and *aac(3)‑IIe*), while other resistance elements were more prone to mosaicism (e.g., *qnrB1*). Nevertheless, a degree of maintenance can be seen for all resistance elements, since these contained one or two dominant mosaic variants which accounted for more than half of the occurrences. Furthermore, IS*26* appeared typically associated with the majority of the mosaic variants that were identified.

Thirdly, we aimed to analyze frequent configurations between these 14 ARG by mapping the flanking regions of the resistance elements. There was evident modularity that ensures that no uniform ARG configuration can be seen in the various MRR. Still, there were certain resistance elements that frequently flanked each other: *bla*_CTX‑M‑15_, *bla*_TEM‑1b_ and *sul2—strA—strB*; *dfrA14*, *qnrB1* and *tet*(A); or *aac(3)‑IIe* and *aac(6’)‑Ib‑cr*—*bla*_OXA‑1_—Δ*catB3*. Hypothetically, these three groups could serve as screening targets during outbreaks of MDR *Enterobacteriaceae* harboring these co-occurring ARGs; however, further empirical evidence is required to validate their practical utility in epidemiological surveillance.

These findings are in line with previous works on the characteristics of IS*26* elements. First, the ARGs were organized into compact MRR of ~20‑50 kb, which appeared to consist of arrayed IS*26* PCTs and IS*26-*associated elements. The variation in flanking regions between the resistance elements could be explained by relocation of translocatable units via conservative transposition to efficiently form various configurations of overlapping PCTs [23]. In addition, 20% of the sequences in this study presented up to five copies of an ARG (both identical and with mosaic variation). It is possible that amplification of translocatable units is involved in this observation. Phenotypically, the elevated expression of the ARG in these duplicate regions could lead to increased minimum inhibitory concentrations against an otherwise effective therapeutic (i.e., carbapenems or amikacin) as previously shown with *bla*_CTX‑M‑15_ and *aac(6’)‑Ib‑cr*—*bla*_OXA‑1_—Δ*catB3* [13,74].

The frequent occurrence of the resistance element *aac(6’)‑Ib‑cr*—*bla*_OXA-1_—Δ*catB3* flanked by two inversely orientated IS*26* elements was remarkable since it should not be able to transpose via the efficient targeted conservative route. This region could potentially function as an anchor for other IS*26* PCTs to transpose towards. This might explain the comparatively high variety of ARG which flanks this region. However, it does not necessarily mean that this resistant element is completely immobile, as it could be integrated in a larger PCT.

Another remarkable finding was the infrequent occurrence of the *aadA1*—*catA1* ARG. Although it was consistently found adjacent to the *sul2*—*strA*—*strB* element, it was also located on the periphery of the various MRR. Similarly, the *tet*(A) variant V5358 appeared associated with a different source organism and capture mechanism than the common Tn*1721* derivates [75,76,81]. Since both the *aadA1*–*catA1* variants and *tet*(A) V5358 were frequently reported in *Enterobacter* spp., it is likely that these resistance elements are limited to a certain plasmid type such as IncHI2 [15,28,34,36,57,85]. Similarly, the dominant occurrence of certain resistance element variants may also be linked to certain plasmid types in *K. pneumoniae* complex (e.g., IncFII(K) and IncFIB) [86], which was the predominant host species in this study. The latter could be the result of a literature bias introduced by a heightened relevance due to the associated pathogenicity and mortality. For instance, most articles included reports on outbreaks, nosocomial transmissions, or severe infections with multi- or extensively drug-resistant isolates that also show resistance to carbapenem or other last-line antimicrobials. Indeed, the dataset of this study frequently contains isolates carrying carbapenemase genes, although rarely in the same plasmid as the 14 ARG examined in this study. Both resistance determinants are relevant to monitor, since each heavily limits clinical treatment options. Independent horizontal transmission of each resistance determinant through unique plasmids may complicate the monitoring in a nosocomial setting [33,41,51]. Still, there also remains a potential that recombination events lead to accumulation of carbapenemase genes and MRR into a single conjugative plasmid [52,55,61].

For infection prevention and control, the practice of outbreak management typically focuses on the transmissions of a single clone which carries identical ARGs (e.g., vancomycin-resistant *Enterococcus*, methicillin-resistant *Staphylococcus aureus* or carbapenemase-producing *Enterobacteriacae*) [3]. However, this approach neglects intercellular transmission of ARGs to other strains and species via plasmids or integrative conjugative elements, but also intracellular transmission of ARG between plasmid and chromosome. Therefore, the characterization of the different nested genetic levels of ARG dissemination (i.e., chromosome, plasmid, and transposons) is necessary to understand an outbreak’s extent. Previous work by Sheppard *et al*. (2016) has demonstrated that the typing of *bla*_KPC_ transposon Tn*4401* in *Enterobacteriaceae* can aid to distinguish between a large outbreak or concurrent smaller outbreaks [4]. The current study also indicates that the characterization of the mosaic variants of certain resistance elements (e.g., *qnrB1*) could hypothetically also provide additional differentiation required to understand the scale of an outbreak.

This study has limitations which should be elaborated upon. The generalizability of dominant resistance element variants should be interpreted with care, as the study design is inherently subject to a selection bias. The collected sequence data is a snapshot of the available literature over the past decade, wherein the use of long-read approaches has been limited compared to short-read approaches. This has made the current study prone to overrepresentation by well-sampled bacterial taxa (i.e., *K. pneumoniae* complex) or successful plasmid lineages which carry recurrent variants, rather than those variants being widely distributed among *Enterobacteriaceae* species. Selection of highly related sequences from outbreak studies has for instance inflated the frequency of certain recurrent variants to an extent. Still, the dominant variants of all resistance element were derived from multiple independent studies with an absolute minimum of seven studies (observed for *aadA1* – *catA1* v5317). Another factor to consider is that the literature search strategy (three to five predefined ARG) combined with the sequence selection criteria (at least six co-occurring ARG) may have essentially favored certain combinations of resistance elements with canonical structures. This may have similarly biased the overrepresentation of certain variants in the dataset. In addition, although the dataset is a selection from various parts of the world, there are still various geographical blind spots. However, an increasing number of isolates will be sequenced using long-read approaches in the coming years. These gaps will be eventually filled, and more clarity will be provided about the role and distribution of MRRs with these co-occurring ARG. Thus, this overview provides a first basis for comparing future sequences. This study did not analyze SNP-variants between the structurally identical resistance elements. However, it may be relevant to further subtype these resistance elements with SNP-analysis tools. This may especially be of interest if applied to a more confined scenario such as a hospital outbreak with similar MRR [12,87]. The current study focused on the modular structure of the involved MRR, but it does not touch upon its mobility. The frequent integration in plasmids and different isolate sequence types indicate that it is potentially spread by means of plasmid conjugation. The typing of the plasmid sequences may elucidate whether the MRR that include these ARG of interest are capable of inter-cellular mobility. This could also provide a better insight into the high rate of *K. pneumoniae* complex in the current dataset, and if plasmid lineage specific mosaic variants can be identified [88].

In conclusion, this study demonstrates that the 14 co-occurring ARG which confer resistance to first- and second-line antimicrobials in *Enterobacteriaceae* is not specific to Dutch outbreaks, but occur globally in MRR with recurrent resistance element structures across diverse genomic contexts. However, the modularity and mosaicism of these associated MRR makes detection and interpretation complex. Still, the preservation of these clinically relevant resistance elements infers a potential means of transfer between distinct *Enterobacteriaceae* isolates. Further analysis of the plasmid sequences is required to elucidate this means.

## Supporting information

S1 Table Search queries in Scholar and PubMed Central. (DOCX)

S2 Table Selected sequences from peer-reviewed literature. Abbreviations: Plasmid sequence (P); Chromosome sequence (C). The asterisk followed by a number indicates plasmids and chromosomes derived from the same isolate.
(DOCX)

S3 Table Isolate data, ARG and mosaic structural variants of resistance elements of included sequences. (XLSX)

S4 Table Post-hoc sensitivity analysis using different ARG thresholds for retention of structural mosaic variants. (XLSX)

S5 Table Frequency of regions located downstream or upstream of the resistance elements. (DOCX)

S1 Fig Heatmap of all ARG found in the literature sequences. The presence of the 14 ARG of interest are visualized by a red box. The presence of the other ARG is visualized by a blue box. The accession of chromosomal sequences are shaded gray, while plasmid sequences remain unshaded.
(PDF)

S2 Fig Genetic structures of all mosaic structural variants. The red arrows indicate ARG. The green arrows indicate IS- and Tn-elements. The blue arrows indicate integron integrase, attI, attC and promotor sites. Arrows with a serrated end present annotations of truncated sequences. The purple arrows indicate complete terminal inverted repeats of a truncated IS*-* or Tn-elements.
(PDF)
