# Supplementary material for: Structural insights into the mosaic and modular organization of fourteen co-occurring resistance genes in multidrug resistant Enterobacteriaceae
Source: PLoS One. 2026 Aug 26;21(8):e0355393. doi: 10.1371/journal.pone.0355393 (PMC13514068; doi:10.1371/journal.pone.0355393)
Supplement: Supplementary file 1 [file pone.0355393.s006.pdf]

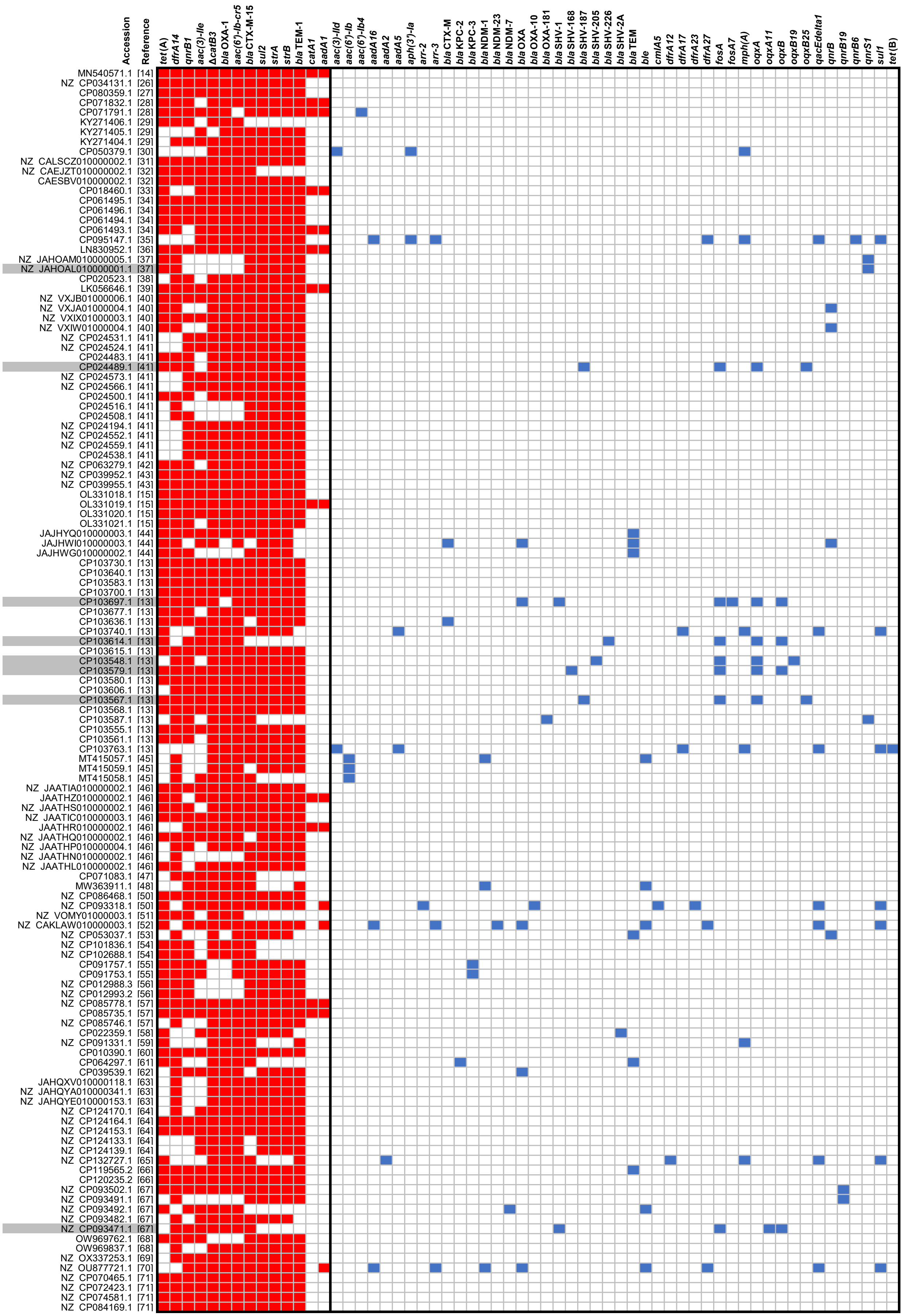
Accession
Reference
tet(A)
dfrA14
qnrB1
aac(3)-IIe
ΔcatB3
bla OXA-1
aac(6')-Ib-cr5
bla CTX-M-15
sul2
strA
strB
bla TEM-1
catA1
aadA1
aac(3)-IId
aac(6')-Ib
aac(6')-Ib4
aadA16
aadA2
aadA5
aph(3')-Ia
arr-2
arr-3
bla CTX-M
bla KPC-2
bla KPC-3
bla NDM-1
bla NDM-23
bla NDM-7
bla OXA
bla OXA-10
bla OXA-181
bla SHV-1
bla SHV-168
bla SHV-187
bla SHV-205
bla SHV-226
bla SHV-2A
bla TEM
ble
cmlA5
dfrA12
dfrA17
dfrA23
dfrA27
fosA
fosA7
mph(A)
oqxA
oqxA11
oqxB
oqxB19
oqxB25
qacEdelta1
qnrB
qnrB19
qnrB6
qnrS1
sul1
tet(B)
MN540571.1 [14]
NZ CP034131.1 [26]
CP080359.1 [27]
CP071832.1 [28]
CP071791.1 [28]
KY271406.1 [29]
KY271405.1 [29]
KY271404.1 [29]
CP050379.1 [30]
NZ CALSCZ010000002.1 [31]
NZ CAEJZT010000002.1 [32]
CAESBV010000002.1 [32]
CP018460.1 [33]
CP061495.1 [34]
CP061496.1 [34]
CP061494.1 [34]
CP061493.1 [34]
CP095147.1 [35]
LN830952.1 [36]
NZ JAHOAM010000005.1 [37]
NZ JAHOAL010000001.1 [37]
CP020523.1 [38]
LK056646.1 [39]
NZ VXJB01000006.1 [40]
NZ VXJA01000004.1 [40]
NZ VXIX01000003.1 [40]
NZ VXIW01000004.1 [40]
NZ CP024531.1 [41]
NZ CP024524.1 [41]
CP024483.1 [41]
CP024489.1 [41]
NZ CP024573.1 [41]
NZ CP024566.1 [41]
CP024500.1 [41]
CP024516.1 [41]
CP024508.1 [41]
NZ CP024194.1 [41]
NZ CP024552.1 [41]
NZ CP024559.1 [41]
CP024538.1 [41]
NZ CP063279.1 [42]
NZ CP039952.1 [43]
NZ CP039955.1 [43]
OL331018.1 [15]
OL331019.1 [15]
OL331020.1 [15]
OL331021.1 [15]
JAJHYQ010000003.1 [44]
JAJHWI010000003.1 [44]
JAJHWG010000002.1 [44]
CP103730.1 [13]
CP103640.1 [13]
CP103583.1 [13]
CP103700.1 [13]
CP103697.1 [13]
CP103677.1 [13]
CP103636.1 [13]
CP103740.1 [13]
CP103614.1 [13]
CP103615.1 [13]
CP103548.1 [13]
CP103579.1 [13]
CP103580.1 [13]
CP103606.1 [13]
CP103567.1 [13]
CP103568.1 [13]
CP103587.1 [13]
CP103555.1 [13]
CP103561.1 [13]
CP103763.1 [13]
MT415057.1 [45]
MT415059.1 [45]
MT415058.1 [45]
NZ JAATIA010000002.1 [46]
JAATHZ010000002.1 [46]
NZ JAATHS010000002.1 [46]
NZ JAATIC010000003.1 [46]
JAATHR010000002.1 [46]
NZ JAATHQ010000002.1 [46]
NZ JAATHP010000004.1 [46]
NZ JAATHN010000002.1 [46]
NZ JAATHL010000002.1 [46]
CP071083.1 [47]
MW363911.1 [48]
NZ CP086468.1 [50]
NZ CP093318.1 [50]
NZ VOMY01000003.1 [51]
NZ CAKLAW010000003.1 [52]
NZ CP053037.1 [53]
NZ CP101836.1 [54]
NZ CP102688.1 [54]
CP091757.1 [55]
CP091753.1 [55]
NZ CP012988.3 [56]
NZ CP012993.2 [56]
NZ CP085778.1 [57]
CP085735.1 [57]
NZ CP085746.1 [57]
CP022359.1 [58]
NZ CP091331.1 [59]
CP010390.1 [60]
CP064297.1 [61]
CP039539.1 [62]
JAHQXV010000118.1 [63]
NZ JAHQYA010000341.1 [63]
NZ JAHQYE010000153.1 [63]
NZ CP124170.1 [64]
NZ CP124164.1 [64]
NZ CP124153.1 [64]
NZ CP124133.1 [64]
NZ CP124139.1 [64]
NZ CP132727.1 [65]
CP119565.2 [66]
CP120235.2 [66]
NZ CP093502.1 [67]
NZ CP093491.1 [67]
NZ CP093492.1 [67]
NZ CP093482.1 [67]
NZ CP093471.1 [67]
OW969762.1 [68]
OW969837.1 [68]
NZ OX337253.1 [69]
NZ OU877721.1 [70]
NZ CP070465.1 [71]
NZ CP072423.1 [71]
NZ CP074581.1 [71]
NZ CP084169.1 [71]
