# Supplementary material for: Structural insights into the mosaic and modular organization of fourteen co-occurring resistance genes in multidrug resistant Enterobacteriaceae
Source: PLoS One. 2026 Aug 26;21(8):e0355393. doi: 10.1371/journal.pone.0355393 (PMC13514068; doi:10.1371/journal.pone.0355393)
Supplement: Supplementary file 2 [file pone.0355393.s007.pdf]

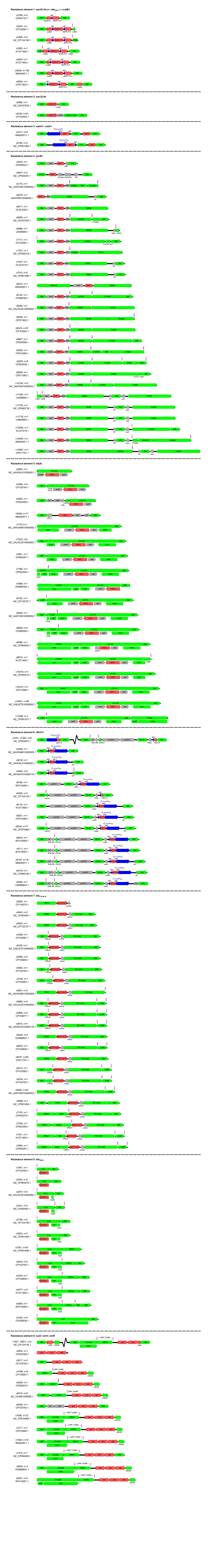

Resistance element 5: tet(A)
Resistance element 6: dfrA14
Resistance element 7: $bla_{CTX-M-15}$
Resistance element 8: bla_{TEM-1}
Resistance element 9: sul2—strA—strB
