# Supplementary material for: Structural insights into the mosaic and modular organization of fourteen co-occurring resistance genes in multidrug resistant Enterobacteriaceae
Source: PLoS One. 2026 Aug 26;21(8):e0355393. doi: 10.1371/journal.pone.0355393 (PMC13514068; doi:10.1371/journal.pone.0355393)
Supplement: Supplementary file 3 [file pone.0355393.s001.docx]

**S1 Table.** Search queries in Scholar and PubMed Central

| **Query number** | **Antimicrobial resistance genes** | **Sequencing method** | **Search query** |
| --- | --- | --- | --- |
| 1 | qnrB1, tetA, dfrA14 | WGS | qnrB1 AND tetA AND dfrA14 AND WGS |
| 2 | aac(6’)-Ib-cr, aac(3)-IIe, OXA-1, catB3 | WGS | aac(6’)-Ib-cr AND aac(3)-IIe AND OXA-1 AND catB3 AND WGS |
| 3 | CTX-M-15, sul2, aph(3")-Ib, aph(6)-Id, catA1 | WGS | CTX-M-15 AND sul2 AND aph(3")-Ib AND aph(6)-Id AND catA1 AND WGS |
| 4 | qnrB1, aac(6’)-Ib-cr, OXA-1, CTX-M-15 | WGS | qnrB1 AND aac(6’)-Ib-cr AND OXA-1 AND CTX-M-15 AND WGS |
| 5 | qnrB1, tetA, dfrA14 | Nanopore | qnrB1 AND tetA AND dfrA14 AND Nanopore |
| 6 | aac(6’)-Ib-cr, aac(3)-IIe, OXA-1, catB3 | Nanopore | aac(6’)-Ib-cr AND aac(3)-IIe AND OXA-1 AND catB3 AND Nanopore |
| 7 | CTX-M-15, sul2, aph(3")-Ib, aph(6)-Id, catA1 | Nanopore | CTX-M-15 AND sul2 AND aph(3")-Ib AND aph(6)-Id AND catA1 AND Nanopore |
| 8 | qnrB1, aac(6’)-Ib-cr, OXA-1, CTX-M-15 | Nanopore | qnrB1 AND aac(6’)-Ib-cr AND OXA-1 AND CTX-M-15 AND Nanopore |
| 9 | qnrB1, tetA, dfrA14 | Pacific Biosciences | qnrB1 AND tetA AND dfrA14 AND Pacific Biosciences |
| 10 | aac(6’)-Ib-cr, aac(3)-IIe, OXA-1, catB3 | Pacific Biosciences | aac(6’)-Ib-cr AND aac(3)-IIe AND OXA-1 AND catB3 AND Pacific Biosciences |
| 11 | CTX-M-15, sul2, aph(3")-Ib, aph(6)-Id, catA1 | Pacific Biosciences | CTX-M-15 AND sul2 AND aph(3")-Ib AND aph(6)-Id AND catA1 AND Pacific Biosciences |
| 12 | qnrB1, aac(6’)-Ib-cr, OXA-1, CTX-M-15 | Pacific Biosciences | qnrB1 AND aac(6’)-Ib-cr AND OXA-1 AND CTX-M-15 AND Pacific Biosciences |
