# Supplementary material for: Structural insights into the mosaic and modular organization of fourteen co-occurring resistance genes in multidrug resistant Enterobacteriaceae
Source: PLoS One. 2026 Aug 26;21(8):e0355393. doi: 10.1371/journal.pone.0355393 (PMC13514068; doi:10.1371/journal.pone.0355393)
Supplement: Supplementary file 4 [file pone.0355393.s002.docx]

**S2 Table.** Selected sequences from peer-reviewed literature.

| Nr. | Author | Species | Host | Location | Year | Sequence | GenBank Accession |
| --- | --- | --- | --- | --- | --- | --- | --- |
| 1 | Marchetti *et al.* [14] | *Escherichia coli* | Dog | Italy | 2017 | P | MN540571.1 |
| 2 | Brinkac *et al.* [26] | *Klebsiella quasipneumoniae* | Human | Nigeria | 2016 | P | NZ_CP034131.1 |
| 3 | Zhang *et al.* [27] | *Klebsiella pneumoniae* | Human | China | 2019 | P | CP080359.1 |
| 4 | Hadjadj *et al.* [28] | *Enterobacter hormachei* | Human | France | 2018 | P | CP071832.1 |
| 5 | Hadjadj *et al.* [28] | *Enterobacter cloacae* | Human | France | 2018 | P | CP071791.1 |
| 6 | Villa *et al.* [29] | *Klebsiella pneumoniae* | Human | United Kingdom | 2015 | P | KY271406.1 |
| 7 | Villa *et al.* [29] | *Klebsiella pneumoniae* | Human | United Kingdom | 2015 | P | KY271405.1 |
| 8 | Villa *et al.* [29] | *Klebsiella pneumoniae* | Human | Italy | 2014 | P | KY271404.1 |
| 9 | Chudejova *et al.* [30] | *Klebsiella pneumoniae* | Human | Czechia | 2018 | P | CP050379.1 |
| 10 | Shapovalova *et al.* [31] | *Klebsiella pneumoniae* | Human | Russia | 2017 | P | NZ_CALSCZ010000002.1 |
| 11 | Baron *et al.* [32] | *Klebsiella pneumoniae* | Chimpanzee | Senegal | 2016 | P | NZ_CAEJZT010000002.1 |
| 12 | Baron *et al.* [32] | *Klebsiella pneumoniae* | Environment | Senegal | 2019 | P | CAESBV010000002.1 |
| 13 | Schwanbeck *et al.* [33] | *Klebsiella pneumoniae* | Human | Germany | 2014 | P | CP018460.1 |
| 14 | Pot *et al.* [34] | *Enterobacter xiangfangensis* | Human | Guadeloupe | 2018 | P | CP061495.1 |
| 15 | Pot *et al.* [34] | *Enterobacter steigerwaltii* | Rat | Guadeloupe | 2018 | P | CP061496.1 |
| 16 | Pot *et al.* [34] | *Enterobacter xiangfangensis* | Goose | Guadeloupe | 2018 | P | CP061494.1 |
| 17 | Pot *et al.* [34] | *Enterobacter xiangfangensis* | Pig | Guadeloupe | 2018 | P | CP061493.1 |
| 18 | Pankok *et al.* [35] | *Klebsiella pneumoniae* | Human | Ghana | 2016 | P | CP095147.1 |
| 19 | Pati *et al.* [36] | *Enterobacter bugandensis* | Human | Tanzania | 2010 | P | LN830952.1 |
| 20 | Abdallah *et al.* [37] | *Escherichia coli* | Green monkey | Senegal | 2019 | P | NZ_JAHOAM010000005.1 |
| 21 | Abdallah *et al.* [37] | *Escherichia coli* | Green monkey | Senegal | 2019 | C | NZ_JAHOAL010000001.1 |
| 22 | Pecora *et al.* [38] | *Escherichia coli* | Human | United States | 2016 | P | CP020523.1 |
| 23 | Feasey *et al.* [39] | *Salmonella enterica serovar Typhimurium* | Human | Malawi | 2009 | P | LK056646.1 |
| 24 | Kopotsa *et al.* [40] | *Klebsiella pneumoniae* | Human | South Africa | 2018 | P | NZ_VXJB01000006.1 |
| 25 | Kopotsa *et al.* [40] | *Klebsiella pneumoniae* | Human | South Africa | 2018 | P | NZ_VXJA01000004.1 |
| 26 | Kopotsa *et al.* [40] | *Klebsiella pneumoniae* | Human | South Africa | 2018 | P | NZ_VXIX01000003.1 |
| 27 | Kopotsa *et al.* [40] | *Klebsiella pneumoniae* | Human | South Africa | 2018 | P | NZ_VXIW01000004.1 |
| 28 | Gorrie *et al.* [41] | *Klebsiella pneumoniae* | Human | Australia | 2013 | P | NZ_CP024531.1 |
| 29 | Gorrie *et al.* [41] | *Klebsiella pneumoniae* | Human | Australia | 2013 | P | NZ_CP024524.1 |
| 30 | Gorrie *et al.* [41] | *Klebsiella pneumoniae* | Human | Australia | 2014 | P | CP024483.1 |
| 31 | Gorrie *et al.* [41] | *Klebsiella pneumoniae* | Human | Australia | 2013 | C | CP024489.1 |
| 32 | Gorrie *et al.* [41] | *Klebsiella pneumoniae* | Human | Australia | 2014 | P | NZ_CP024573.1 |
| 33 | Gorrie *et al.* [41] | *Klebsiella pneumoniae* | Human | Australia | 2014 | P | NZ_CP024566.1 |
| 34 | Gorrie *et al.* [41] | *Klebsiella pneumoniae* | Human | Australia | 2013 | P | CP024500.1 |
| 35 | Gorrie *et al.* [41] | *Klebsiella pneumoniae* | Human | Australia | 2014 | P | CP024516.1 |
| 36 | Gorrie *et al.* [41] | *Klebsiella pneumoniae* | Human | Australia | 2013 | P | CP024508.1 |
| 37 | Gorrie *et al.* [41] | *Klebsiella pneumoniae* | Human | Australia | 2013 | P | NZ_CP024194.1 |
| 38 | Gorrie *et al.* [41] | *Klebsiella pneumoniae* | Human | Australia | 2013 | P | NZ_CP024552.1 |
| 39 | Gorrie *et al.* [41] | *Klebsiella pneumoniae* | Human | Australia | 2014 | P | NZ_CP024559.1 |
| 40 | Gorrie *et al.* [41] | *Klebsiella pneumoniae* | Human | Australia | 2013 | P | CP024538.1 |
| 41 | Shaidullina *et al.* [42] | *Klebsiella pneumoniae* | Human | Russia | 2014 | P | NZ_CP063279.1 |
| 42 | Brilhante *et al.* [43] | *Klebsiella pneumoniae* | Dog | Switzerland | 2018 | P | NZ_CP039952.1 |
| 43 | Brilhante *et al.* [43] | *Klebsiella pneumoniae* | Cat | Switzerland | 2018 | P | NZ_CP039955.1 |
| 44 | Pot *et al.* [15] | *Enterobacter hormaechei* | Human | Guadeloupe | 2018 | P | OL331018.1 |
| 45 | Pot *et al.* [15] | *Enterobacter asburiae* | Human | Guadeloupe | 2019 | P | OL331019.1 |
| 46 | Pot *et al.* [15] | *Enterobacter cloacae* | Human | Guadeloupe | 2019 | P | OL331020.1 |
| 47 | Pot *et al.* [15] | *Enterobacter cloacae* | Human | Guadeloupe | 2019 | P | OL331021.1 |
| 48 | Muraya *et al.* [44] | *Klebsiella pneumoniae* | Human | Kenya | 2019 | P | JAJHYQ010000003.1 |
| 49 | Muraya *et al.* [44] | *Klebsiella pneumoniae* | Environment | Kenya | NA | P | JAJHWI010000003.1 |
| 50 | Muraya *et al.* [44] | *Klebsiella pneumoniae* | Environment | Kenya | NA | P | JAJHWG010000002.1 |
| 51 | Shropshire *et al.* [13] | *Klebsiella pneumoniae* | Human | United States | 2016 | P | CP103730.1 |
| 52 | Shropshire *et al.* [13] | *Klebsiella aerogenes* | Human | United States | 2018 | P | CP103640.1 |
| 53 | Shropshire *et al.* [13] | *Klebsiella pneumoniae* | Human | United States | 2019 | P | CP103583.1 |
| 54 | Shropshire *et al.* [13] | *Klebsiella pneumoniae* | Human | United States | 2017 | P | CP103700.1 |
| 55 | Shropshire *et al.* [13] | *Klebsiella pneumoniae* | Human | United States | 2017 | C | CP103697.1 |
| 56 | Shropshire *et al.* [13] | *Klebsiella pneumoniae* | Human | United States | 2017 | P | CP103677.1 |
| 57 | Shropshire *et al.* [13] | *Klebsiella pneumoniae* | Human | United States | 2018 | P | CP103636.1 |
| 58 | Shropshire *et al.* [13] | *Escherichia coli* | Human | United States | 2016 | P | CP103740.1 |
| 59 | Shropshire *et al.* [13] | *Klebsiella pneumoniae* | Human | United States | 2018 | C*1 | CP103614.1 |
| 60 | Shropshire *et al.* [13] | *Klebsiella pneumoniae* | Human | United States | 2018 | P*1 | CP103615.1 |
| 61 | Shropshire *et al.* [13] | *Klebsiella pneumoniae* | Human | United States | 2019 | C | CP103548.1 |
| 62 | Shropshire *et al.* [13] | *Klebsiella pneumoniae* | Human | United States | 2019 | C*2 | CP103579.1 |
| 63 | Shropshire *et al.* [13] | *Klebsiella pneumoniae* | Human | United States | 2019 | P*2 | CP103580.1 |
| 64 | Shropshire *et al.* [13] | *Klebsiella pneumoniae* | Human | United States | 2018 | P | CP103606.1 |
| 65 | Shropshire *et al.* [13] | *Klebsiella pneumoniae* | Human | United States | 2019 | C*3 | CP103567.1 |
| 66 | Shropshire *et al.* [13] | *Klebsiella pneumoniae* | Human | United States | 2019 | P*3 | CP103568.1 |
| 67 | Shropshire *et al.* [13] | *Klebsiella pneumoniae* | Human | United States | 2019 | P | CP103587.1 |
| 68 | Shropshire *et al.* [13] | *Klebsiella michiganensis* | Human | United States | 2019 | P | CP103555.1 |
| 69 | Shropshire *et al.* [13] | *Klebsiella pneumoniae* | Human | United States | 2019 | P | CP103561.1 |
| 70 | Shropshire *et al.* [13] | *Escherichia coli* | Human | United States | 2020 | P | CP103763.1 |
| 71 | Izdebski *et al.* [45] | *Klebsiella pneumoniae* | Human | Poland | 2012 | P | MT415057.1 |
| 72 | Izdebski *et al.* [45] | *Klebsiella pneumoniae* | Human | Poland | 2013 | P | MT415059.1 |
| 73 | Izdebski *et al.* [45] | *Klebsiella pneumoniae* | Human | Poland | 2013 | P | MT415058.1 |
| 74 | Evans *et al.* [46] | *Citrobacter braakii* | Human | United States | 2017 | P | NZ_JAATIA010000002.1 |
| 75 | Evans *et al.* [46] | *Citrobacter freundii* | Human | United States | 2017 | P | JAATHZ010000002.1 |
| 76 | Evans *et al.* [46] | *Klebsiella pneumoniae* | Human | United States | 2017 | P | NZ_JAATHS010000002.1 |
| 77 | Evans *et al.* [46] | *Klebsiella quasipneumoniae* | Human | United States | 2017 | P | NZ_JAATIC010000003.1 |
| 78 | Evans *et al.* [46] | *Klebsiella pneumoniae* | Human | United States | 2017 | P | JAATHR010000002.1 |
| 79 | Evans *et al.* [46] | *Klebsiella pneumoniae* | Human | United States | 2017 | P | NZ_JAATHQ010000002.1 |
| 80 | Evans *et al.* [46] | *Klebsiella pneumoniae* | Human | United States | 2017 | P | NZ_JAATHP010000004.1 |
| 81 | Evans *et al.* [46] | *Klebsiella pneumoniae* | Human | United States | 2017 | P | NZ_JAATHN010000002.1 |
| 82 | Evans *et al.* [46] | *Serratia marcescens* | Human | United States | 2017 | P | NZ_JAATHL010000002.1 |
| 83 | Moser *et al.* [47] | *Klebsiella pneumoniae* | Human | Switzerland | 2020 | P | CP071083.1 |
| 84 | Biedrzycka *et al.* [48] | *Klebsiella pneumoniae* | Human | Poland | 2019 | P | MW363911.1 |
| 85 | Moser *et al.* [49] | *Klebsiella pneumoniae* | Human | Switzerland | 2017 | P | NZ_CP086468.1 |
| 86 | Rahube *et al.* [50] | *Escherichia coli* | Human | Botswana | 2012 | P | NZ_CP093318.1 |
| 87 | Chen *et al.* [51] | *Klebsiella pneumoniae* | Human | Singapore | 2014 | P | NZ_VOMY01000003.1 |
| 88 | García-González *et al.* [52] | *Klebsiella pneumoniae* | Human | Spain | 2016 | P | NZ_CAKLAW010000003.1 |
| 89 | Martins *et al.* [53] | *Klebsiella pneumoniae* | Human | Brazil | 2015 | P | NZ_CP053037.1 |
| 90 | Stone *et al.* [54] | *Klebsiella pneumoniae* | Human | Jamaica | 2021 | P | NZ_CP101836.1 |
| 91 | Stone *et al.* [54] | *Klebsiella pneumoniae* | Human | Jamaica | 2021 | P | NZ_CP102688.1 |
| 92 | Campos‑Madueno *et al.* [55] | *Escherichia coli* | Human | Italy | 2020 | P | CP091757.1 |
| 93 | Campos‑Madueno *et al.* [55] | *Klebsiella grimontii* | Human | Italy | 2020 | P | CP091753.1 |
| 94 | Lynch *et al.* [56] | *Klebsiella pneumoniae* | Human | Canada | 2013 | P | NZ_CP012988.3 |
| 95 | Lynch *et al.* [56] | *Klebsiella pneumoniae* | Human | Canada | 2013 | P | NZ_CP012993.2 |
| 96 | Bitar *et al.* [57] | *Enterobacter hormachei* | Human | Czechia | 2019 | P | NZ_CP085778.1 |
| 97 | Bitar *et al.* [57] | *Enterobacter cloacae* | Human | Czechia | 2020 | P | CP085735.1 |
| 98 | Bitar *et al.* [57] | *Enterobacter hormachei* | Human | Czechia | 2020 | P | NZ_CP085746.1 |
| 99 | Jousset *et al.* [58] | *Shewanella sp.* | Human | France | 2015 | P | CP022359.1 |
| 100 | Wang *et al.* [59] | *Klebsiella pneumoniae* | Human | China | 2018 | P | NZ_CP091331.1 |
| 101 | Rojas *et al.* [60] | *Klebsiella pneumoniae* | Human | Colombia | 2012 | P | CP010390.1 |
| 102 | Abril *et al.* [61] | *Klebsiella pneumoniae* | Human | Colombia | 2015 | P | CP064297.1 |
| 103 | Su *et al.* [62] | *Klebsiella pneumoniae* | Human | China | 2017 | P | CP039539.1 |
| 104 | Perez-Palacios *et al.* [63] | *Klebsiella pneumoniae* | Human | Spain | 2012 | P | JAHQXV010000118.1 |
| 105 | Perez-Palacios *et al.* [63] | *Klebsiella pneumoniae* | Human | Spain | 2018 | P | NZ_JAHQYA010000341.1 |
| 106 | Perez-Palacios *et al.* [63] | *Klebsiella pneumoniae* | Human | Spain | 2018 | P | NZ_JAHQYE010000153.1 |
| 107 | Lascols *et al.* [64] | *Klebsiella pneumoniae* | Human | Pakistan | 2013 | P | NZ_CP124170.1 |
| 108 | Lascols *et al.* [64] | *Klebsiella pneumoniae* | Human | Pakistan | 2013 | P | NZ_CP124164.1 |
| 109 | Lascols *et al.* [64] | *Klebsiella pneumoniae* | Human | Pakistan | 2013 | P | NZ_CP124153.1 |
| 110 | Lascols *et al.* [64] | *Klebsiella pneumoniae* | Human | Pakistan | 2013 | P | NZ_CP124133.1 |
| 111 | Lascols *et al.* [64] | *Klebsiella pneumoniae* | Human | Pakistan | 2013 | P | NZ_CP124139.1 |
| 112 | Li *et al.* [65] | *Klebsiella pneumoniae* | Environment | China | 2021 | P | NZ_CP132727.1 |
| 113 | Fordham *et al.* [66] | *Klebsiella pneumoniae* | Human | United Kingdom | 2022 | P | CP119565.2 |
| 114 | Fordham *et al.* [66] | *Escherichia coli* | Human | United Kingdom | 2022 | P | CP120235.2 |
| 115 | Veloso *et al.* [67] | *Klebsiella pneumoniae* | Human | Chile | 2018 | P | NZ_CP093502.1 |
| 116 | Veloso *et al.* [67] | *Klebsiella pneumoniae* | Human | Chile | 2019 | P*4 | NZ_CP093491.1 |
| 117 | Veloso *et al.* [67] | *Klebsiella pneumoniae* | Human | Chile | 2019 | P*4 | NZ_CP093492.1 |
| 118 | Veloso *et al.* [67] | *Klebsiella pneumoniae* | Human | Chile | 2019 | P | NZ_CP093482.1 |
| 119 | Veloso *et al.* [67] | *Klebsiella pneumoniae* | Human | Chile | 2019 | C | NZ_CP093471.1 |
| 120 | Gato *et al.* [68] | Klebsiella pneumoniae | Human | Spain | 2016 | P | OW969762.1 |
| 121 | Gato *et al.* [68] | Klebsiella pneumoniae | Human | Spain | 2015 | P | OW969837.1 |
| 122 | Mentasti *et al.* [69] | Klebsiella pneumoniae | Human | United Kingdom | 2020 | P | NZ_OX337253.1 |
| 123 | García-González *et al.* [70] | Klebsiella pneumoniae | Human | Spain | 2016 | P | NZ_OU877721.1 |
| 124 | Manesh *et al.* [71] | Klebsiella pneumoniae | Human | India | 2014 | P | NZ_CP070465.1 |
| 125 | Manesh *et al.* [71] | Klebsiella pneumoniae | Human | India | 2014 | P | NZ_CP072423.1 |
| 126 | Manesh *et al.* [71] | Klebsiella pneumoniae | Human | India | 2014 | P | NZ_CP074581.1 |
| 127 | Manesh *et al.* [71] | Klebsiella pneumoniae | Human | India | 2015 | P | NZ_CP084169.1 |

*Abbreviations*: Plasmid sequence (P); Chromosome sequence (C). The asterisk followed by a number indicates plasmids and chromosomes derived from the same isolate.
