# Supplementary material for: Structural insights into the mosaic and modular organization of fourteen co-occurring resistance genes in multidrug resistant Enterobacteriaceae
Source: PLoS One. 2026 Aug 26;21(8):e0355393. doi: 10.1371/journal.pone.0355393 (PMC13514068; doi:10.1371/journal.pone.0355393)
Supplement: Supplementary file 5 [file pone.0355393.s005.docx]

**S4 Table.** Frequency of regions located downstream or upstream of the resistance elements.

|  | *aac(6')-Ib-cr*—*bla*_OXA-1_—  Δ*catB3* (Block 1) | | *aac(3)-IIe* (Block 2) | | *aadA1****—****catA1* (Block 3) | | *qnrB1* (Block 4) | | *tet*(A) (Block 5) | | *dfrA14* (Block 6) | | *bla*_CTX-M-15_ (Block 7) | | *bla*_TEM-1b_ (Block 8) | | *sul2—strA—strB* (Block 9) | |
| --- | --- | --- | --- | --- | --- | --- | --- | --- | --- | --- | --- | --- | --- | --- | --- | --- | --- | --- |
|  | Up | Down | Up | Down | Up | Down | Up | Down | Up | Down | Up | Down | Up | Down | Up | Down | Up | Down |
| *aac(6')-Ib-cr*—*bla*_OXA-1_—Δ*catB3* (Block 1) | 7 | 5 | 74 | 0 | 0 | 0 | 15 | 0 | 54 | 3 | 19 | 7 | 9 | 23 | 4 | 0 | 2 | 0 |
| *aac(3)-IIe* (Block 2) | 4 | 70 | 0 | 0 | 0 | 0 | 1 | 14 | 3 | 0 | 13 | 0 | 0 | 37 | 5 | 0 | 3 | 0 |
| *aadA1*—*catA1* (Block 3) | 0 | 0 | 0 | 0 | 0 | 0 | 0 | 0 | 0 | 0 | 0 | 0 | 0 | 0 | 0 | 0 | 11 | 0 |
| *qnrB1* (Block 4) | 12 | 3 | 13 | 2 | 0 | 0 | 1 | 1 | 0 | 63 | 65 | 8 | 1 | 2 | 1 | 0 | 4 | 0 |
| *tet*(A) (Block 5) | 45 | 12 | 0 | 3 | 0 | 0 | 63 | 0 | 0 | 0 | 3 | 15 | 3 | 0 | 2 | 2 | 2 | 5 |
| *dfrA14* (Block 6) | 12 | 14 | 0 | 13 | 0 | 0 | 8 | 64 | 2 | 17 | 0 | 0 | 0 | 48 | 3 | 0 | 3 | 0 |
| *bla*_CTX-M-15_ (Block 7) | 22 | 11 | 0 | 37 | 0 | 0 | 0 | 3 | 3 | 0 | 0 | 48 | 1 | 7 | 100 | 0 | 1 | 1 |
| *bla*_TEM-1b_ (Block 8) | 2 | 1 | 2 | 3 | 0 | 0 | 1 | 0 | 3 | 1 | 1 | 2 | 99 | 1 | 0 | 0 | 0 | 113 |
| *sul2—strA—strB* (Block 9) | 0 | 2 | 1 | 2 | 11 | 0 | 4 | 0 | 7 | 0 | 2 | 2 | 1 | 1 | 0 | 113 | 0 | 0 |
| Other ARG | 2 | 1 | 0 | 0 | 0 | 0 | 1 | 3 | 0 | 1 | 0 | 0 | 3 | 4 | 0 | 3 | 3 | 1 |
| Non-ARG region | 31 | 18 | 2 | 32 | 1 | 12 | 7 | 16 | 22 | 9 | 20 | 41 | 20 | 14 | 4 | 1 | 93 | 2 |
| Total (*n*=) | 137 | 137 | 92 | 92 | 12 | 12 | 101 | 101 | 94 | 94 | 123 | 123 | 137 | 137 | 119 | 119 | 122 | 122 |

*Note:* The column header indicates the resistance element from which the observation takes place. The column sub-header indicates whether a flanking region is located upstream (Up) or downstream (Down). The column rows indicate the flanking regions. The frequency is presented by the integers. The color of the integer cells represent the relative occurrence of each flanking region; a relatively high occurrence is presented by green cells, while a relatively low occurrence is represented by orange cells.
