# Supplementary material for: Structural insights into the mosaic and modular organization of fourteen co-occurring resistance genes in multidrug resistant Enterobacteriaceae
Source: PLoS One. 2026 Aug 26;21(8):e0355393. doi: 10.1371/journal.pone.0355393 (PMC13514068; doi:10.1371/journal.pone.0355393)
Supplement: Supplementary file 6 [file pone.0355393.s009.docx]

**Response to Reviewers**

**Ref:** PONE-D-26-13574
**Manuscript:** Structural insights into the mosaic and modular organization of fourteen co-occurring resistance genes in multidrug resistant Enterobacteriaceae

Dear reviewer,

We would like to express our thanks for the thorough review and insightful comments on our manuscript. The comments provided were brought forward in a comprehensive and constructive manner which is truly appreciated.

Overall, we agree with the reviewer and have revised the manuscript to improve methodological transparency, clarify analytical decisions and restrain speculative claims.

The pages below describe our point-by-point response to the major and minor comments.

Sincerely,

Connor Rossel

Also on behalf of my co-authors

**Major comments:**

1. Please clarify the methodological nature of the study. The manuscript combines a literature search with downstream comparative genomic analysis, but it is not entirely clear how the study should be classified methodologically. The work has features of a structured review, but it is not reported as a systematic review. This distinction matters because readers need to understand whether the literature identification and screening process was intended to be comprehensive and reproducible. A clearer statement in the Methods would be helpful. A study-selection flow diagram would also improve transparency.

This is a valid remark, which we have clarified further in the method section (line 83).

We think that the nature of the study can be best described as a structured literature review. The label of systematic literature review would not be appropriate. The core objective of the current study is to collect sequences for secondary genomic analysis rather than to critically appraise and synthesize the findings of the prior studies themselves. Also related to this difference in core objective, the current study does not meet other important criteria considered standard for a systematic review, such as risk-of-bias analyses for the included studies and strict use of indexed biomedical databases.

A study-selection flow diagram has also been added to the results section (Fig 1).

2. The search strategy may enrich for the very structures under investigation. Because the literature search was ARG-centric and required combinations of three to five predefined genes, as well as at least six co-occurring ARG in a long-read assembly, the study design may preferentially retrieve highly relevant examples of these multidrug resistance regions while excluding related but less canonical structures. Please discuss more explicitly how this selection strategy may have biased the observed frequency and apparent recurrence of these ARG blocks.

This is indeed a possibility. We described this bias more explicitly together with major comment 5 and minor comment 2 in the discussion (line 398-411).

3. The rationale for selecting a threshold of at least six ARG needs to be strengthened. The cutoff is justified by prior observations in the Netherlands, where six genes were linked to a similar MDR phenotype. However, the present manuscript uses this threshold for a global structural analysis. Please explain more clearly why this threshold is appropriate beyond the original local setting. If feasible, a sensitivity analysis using different thresholds would strengthen confidence that the main findings are not an artefact of this choice.

We chose this specific threshold based on the potential inference of MDR phenotypes against (global cornerstone) antimicrobial categories for the treatment of *Enterobacteriaceae* infections. This includes non-susceptibility to aminoglycosides by *aac(3)-IIe* or *aac(6')-Ib-cr*, to cotrimoxazole by *dfrA14* and *sul2*, to fluoroquinolones by *qnrB1* or *aac(6')-Ib-cr*, and to extended-spectrum β-lactams by *bla*_CTX-M-15_. This has been described in the methods (line 91- 96).

As suggested, we applied a post-hoc analysis for the impact of different thresholds on the main findings (line 278-290, S4 Table). Increasing the threshold appears to decrease genomic sequences and structural mosaic variants in a proportionate manner. The mosaic variants lost during the increase of the threshold are those observed in a low frequency. Increasing thresholds (e.g., to ≥8 ARG) does not appear to change the predominant mosaic variants (n > 10) described in the body of the manuscript. Therefore, we do not expect that the main findings are an artefact of the initial cut-off of 6 ARG.

4. Please justify how the nine ARG blocks were defined. The Methods state that the direct environment of each ARG was analyzed to reduce the influence of broader modular rearrangement, and that tandem ARG were grouped into a single resistance element. However, the criteria used to delimit each block are not fully explicit. Were these boundaries defined manually, by fixed rules, or iteratively after inspection? This needs to be described more rigorously, particularly for regions involving IS26, truncated elements, or tandem resistance genes.

This section has been rewritten in the methods to clarify which considerations were applied in the identification of resistance elements and the demarcation of their boundaries (line 117-146). This includes clarification on regions involving IS26, truncated elements and tandem resistance genes.

5. The dominance of certain taxa and plasmid contexts limits generalizability. Most included sequences were derived from plasmids, and Klebsiella pneumoniae complex was the dominant taxon. This skew should be addressed more directly in the Discussion. It is possible that some of the observed recurrence reflects the repeated recovery of successful plasmid scaffolds or well-sampled lineages rather than broadly distributed architecture across all Enterobacteriaceae.

This bias is indeed a drawback of the currently used approach. We have stated this concern more explicitly in the limitations of the discussion (line 398-404).

6. Claims regarding transfer should be phrased more cautiously. The conclusion states that preservation of these clinically relevant ARG suggests effective transfer of these resistance elements between distinct isolates. This is plausible, but the study does not directly demonstrate transfer, mobility, or conjugation. At most, the data support recurrent structural association across diverse genomic contexts. I recommend softening the conclusion to reflect that inter-isolate transfer is inferred rather than shown.

This was indeed our intention. The sentence has been altered to: “the preservation of these clinically relevant resistance elements infers a potential means of transfer between distinct *Enterobacteriaceae* isolates.”. A follow-up sentence is also added to make this clear: “Further analysis of the plasmid sequences is required to elucidate this means.” (line 429-431)

7. Functional interpretation should distinguish conserved structure from active resistance contribution. The manuscript notes that catB3 was truncated in all instances and does not confer chloramphenicol resistance. This is important. Please make a clearer distinction between loci that are structurally recurrent and loci that remain functionally relevant to phenotype. Otherwise, readers may overinterpret the clinical resistance contribution of all genes within the described blocks.

Indeed, a few elements have recurrent variants with truncated resistance genes which likely do not confer the associated phenotype anymore. Besides the Δ*catB3* that was truncated in all structures, *drfrA14* was truncated in v2791-V7304 (n=8) and v6428 (n=2), and *strB* was truncated in v1921-v6811 (n=2). (line 249-251 and line 276-277).

We have made mentioned of these variants under the section “Mosaicism of the resistance elements” in their respective paragraphs. We chose to mention this here since these resistance genes were not initially detected with AMRFinderPlus (<60% coverage); it was detected during assessment of the mosaic structure of the resistance elements.

8. Assembly quality and annotation uncertainty deserve more attention. The analysis combines public long-read assemblies generated over many years and annotates them using several tools. Please clarify whether any assembly quality thresholds were applied and how disagreements between annotation tools were resolved. This is especially important for IS-rich regions, which are prone to assembly fragmentation and annotation inconsistency.

We did not apply any assembly quality threshold and clarified this in the methods section. (line 100).

A clarification for the resolution of discrepant annotation is also added. (line 112-116).

9. The proposed outbreak application is interesting but currently speculative. The manuscript suggests that recognizing recurrent co-localized ARG could provide potential targets for screening during outbreaks. This should be presented more carefully as a potential future application rather than an evidence-based practical recommendation arising from the current study.

This sentence has been rephrased more cautiously to clearly express the speculative nature. “Hypothetically, these three groups could serve as screening targets during outbreaks of MDR Enterobacteriaceae harboring these co-occurring ARGs; however, further empirical evidence is required to validate their practical utility in epidemiological surveillance.” (line 348-351).

**Minor comments:**

1. Add a study-selection figure. A simple visual workflow for article identification, screening, exclusion, and inclusion would greatly improve readability.

We have added a study selection figure in the results section (Fig 1). (line 160)

2. Clarify whether repeated or highly related sequences may have inflated the frequency of dominant variants. This is particularly relevant when interpreting the most common structural variants.

This has been clarified in the discussion. (line 404-407).

3. Consider summarizing the main characteristics of included sequences in the main manuscript rather than relying heavily on supplementary tables. This would improve accessibility for readers.

Unfortunately, we cannot determine which specific main characteristics of the sequences are meant here. The characteristics found in supplementary table 2 have been summarized in the main body text of the manuscript. This includes geography, host, taxonomy, and type of sequence (plasmids/chromosome). (line 162-173).
We have adjusted the text slightly in the main text to better indicate in advance what supplementary table 2 contains, should a reader be interested in the sequence specific characteristics.

4. It may help to define key terms early, such as "mosaic", "modular", "block", "variant", etc. to avoid ambiguity.

We have made some alterations in the introduction of the manuscript to better define these terms early on.

Throughout the manuscript we have also altered the word “block” into “resistance elements”, as both terms were used interchangeably.
