# Supplementary material for: Structural insights into the mosaic and modular organization of fourteen co-occurring resistance genes in multidrug resistant Enterobacteriaceae
Source: PLoS One. 2026 Aug 26;21(8):e0355393. doi: 10.1371/journal.pone.0355393 (PMC13514068; doi:10.1371/journal.pone.0355393)
Supplement: Peer review history

# Peer review: Decision Letter 0

**DOI:** 10.1371/journal.pone.0355393.r001

**Authors:** Clinton Moodley

20 May 2026

PONE-D-26-13574

Structural insights into the mosaic and modular organization of fourteen co-occurring resistance genes in multidrug resistant Enterobacteriaceae

PLOS One

Dear Dr. Rossel,

Thank you for submitting your manuscript to PLOS ONE. After careful consideration, we feel that it has merit but does not fully meet PLOS ONE’s publication criteria as it currently stands. Therefore, we invite you to submit a revised version of the manuscript that addresses the points raised during the review process.

Please submit your revised manuscript by Jul 04 2026 11:59PM. If you will need more time than this to complete your revisions, please reply to this message or contact the journal office at plosone@plos.org. When you're ready to submit your revision, log on to https://www.editorialmanager.com/pone/ and select the 'Submissions Needing Revision' folder to locate your manuscript file.

Please include the following items when submitting your revised manuscript:

- A letter that responds to each point raised by the academic editor and reviewer(s). You should upload this letter as a separate file labeled 'Response to Reviewers'.
- A marked-up copy of your manuscript that highlights changes made to the original version. You should upload this as a separate file labeled 'Revised Manuscript with Track Changes'.
- An unmarked version of your revised paper without tracked changes. You should upload this as a separate file labeled 'Manuscript'.

If you would like to make changes to your financial disclosure, please include your updated statement in your cover letter. Guidelines for resubmitting your figure files are available below the reviewer comments at the end of this letter.

If applicable, we recommend that you deposit your laboratory protocols in protocols.io to enhance the reproducibility of your results. Protocols.io assigns your protocol its own identifier (DOI) so that it can be cited independently in the future. For instructions see: https://journals.plos.org/plosone/s/submission-guidelines#loc-laboratory-protocols. Additionally, PLOS ONE offers an option for publishing peer-reviewed Lab Protocol articles, which describe protocols hosted on protocols.io. Read more information on sharing protocols at https://plos.org/protocols?utm_medium=editorial-email&utm_source=authorletters&utm_campaign=protocols.

As the corresponding author, your ORCID iD is verified in the submission system and will appear in the published article. PLOS supports the use of ORCID, and we encourage all coauthors to register for an ORCID iD and use it as well. Please encourage your coauthors to verify their ORCID iD within the submission system before final acceptance, as unverified ORCID iDs will not appear in the published article. *Only*  the individual author can complete the verification step; PLOS staff *cannot*  verify ORCID iDs on behalf of authors.

We look forward to receiving your revised manuscript.

Kind regards,

Clinton Moodley, Ph.D.

Academic Editor

PLOS One

Journal Requirements:

When submitting your revision, we need you to address these additional requirements.

1. Please ensure that your manuscript meets PLOS ONE's style requirements, including those for file naming. The PLOS ONE style templates can be found at

https://journals.plos.org/plosone/s/file?id=wjVg/PLOSOne_formatting_sample_main_body.pdf and

https://journals.plos.org/plosone/s/file?id=ba62/PLOSOne_formatting_sample_title_authors_affiliations.pdf

2. Please note that PLOS One has specific guidelines on code sharing for submissions in which author-generated code underpins the findings in the manuscript. In these cases, we expect all author-generated code to be made available without restrictions upon publication of the work. Please review our guidelines at https://journals.plos.org/plosone/s/materials-and-software-sharing#loc-sharing-code and ensure that your code is shared in a way that follows best practice and facilitates reproducibility and reuse.

3. If the reviewer comments include a recommendation to cite specific previously published works, please review and evaluate these publications to determine whether they are relevant and should be cited. There is no requirement to cite these works unless the editor has indicated otherwise.

[Note: HTML markup is below. Please do not edit.]

Reviewers' comments:

Reviewer's Responses to Questions

**Comments to the Author**

1. Is the manuscript technically sound, and do the data support the conclusions?

The manuscript must describe a technically sound piece of scientific research with data that supports the conclusions. Experiments must have been conducted rigorously, with appropriate controls, replication, and sample sizes. The conclusions must be drawn appropriately based on the data presented.

Reviewer #1: Partly

**********

2. Has the statistical analysis been performed appropriately and rigorously?

Reviewer #1: Yes

**********

3. Have the authors made all data underlying the findings in their manuscript fully available?

The PLOS Data policy requires authors to make all data underlying the findings described in their manuscript fully available without restriction, with rare exception (please refer to the Data Availability Statement in the manuscript PDF file). The data should be provided as part of the manuscript or its supporting information, or deposited to a public repository. For example, in addition to summary statistics, the data points behind means, medians and variance measures should be available. If there are restrictions on publicly sharing data—e.g. participant privacy or use of data from a third party—those must be specified.

Reviewer #1: Yes

**********

4. Is the manuscript presented in an intelligible fashion and written in standard English?

PLOS ONE does not copyedit accepted manuscripts, so the language in submitted articles must be clear, correct, and unambiguous. Any typographical or grammatical errors should be corrected at revision, so please note any specific errors here.

Reviewer #1: Yes

**********

5. Review Comments to the Author

Please use the space provided to explain your answers to the questions above. You may also include additional comments for the author, including concerns about dual publication, research ethics, or publication ethics. (Please upload your review as an attachment if it exceeds 20,000 characters)

Reviewer #1: Comments to the authors:

This manuscript addresses an important and timely topic in antimicrobial resistance genomics. The focus on the structural organization of 14 co-occurring resistance genes across multiple Enterobacteriaceae taxa is relevant, and the attempt to analyze resistance-region architecture rather than relying solely on strain clonality is a strength. The use of literature-derived long-read assemblies from multiple geographic regions also adds potential value.

At the same time, the manuscript would benefit from revision to improve methodological transparency, strengthen interpretive restraint, and clarify several key analytical decisions. My main concerns are outlined below.

Major comments:

1. Please clarify the methodological nature of the study. The manuscript combines a literature search with downstream comparative genomic analysis, but it is not entirely clear how the study should be classified methodologically. The work has features of a structured review, but it is not reported as a systematic review. This distinction matters because readers need to understand whether the literature identification and screening process was intended to be comprehensive and reproducible. A clearer statement in the Methods would be helpful. A study-selection flow diagram would also improve transparency.

2. The search strategy may enrich for the very structures under investigation. Because the literature search was ARG-centric and required combinations of three to five predefined genes, as well as at least six co-occurring ARG in a long-read assembly, the study design may preferentially retrieve highly relevant examples of these multidrug resistance regions while excluding related but less canonical structures. Please discuss more explicitly how this selection strategy may have biased the observed frequency and apparent recurrence of these ARG blocks.

3. The rationale for selecting a threshold of at least six ARG needs to be strengthened. The cutoff is justified by prior observations in the Netherlands, where six genes were linked to a similar MDR phenotype. However, the present manuscript uses this threshold for a global structural analysis. Please explain more clearly why this threshold is appropriate beyond the original local setting. If feasible, a sensitivity analysis using different thresholds would strengthen confidence that the main findings are not an artefact of this choice.

4. Please justify how the nine ARG blocks were defined. The Methods state that the direct environment of each ARG was analyzed to reduce the influence of broader modular rearrangement, and that tandem ARG were grouped into a single resistance element. However, the criteria used to delimit each block are not fully explicit. Were these boundaries defined manually, by fixed rules, or iteratively after inspection? This needs to be described more rigorously, particularly for regions involving IS26, truncated elements, or tandem resistance genes.

5. The dominance of certain taxa and plasmid contexts limits generalizability. Most included sequences were derived from plasmids, and Klebsiella pneumoniae complex was the dominant taxon. This skew should be addressed more directly in the Discussion. It is possible that some of the observed recurrence reflects the repeated recovery of successful plasmid scaffolds or well-sampled lineages rather than broadly distributed architecture across all Enterobacteriaceae.

6. Claims regarding transfer should be phrased more cautiously. The conclusion states that preservation of these clinically relevant ARG suggests effective transfer of these resistance elements between distinct isolates. This is plausible, but the study does not directly demonstrate transfer, mobility, or conjugation. At most, the data support recurrent structural association across diverse genomic contexts. I recommend softening the conclusion to reflect that inter-isolate transfer is inferred rather than shown.

7. Functional interpretation should distinguish conserved structure from active resistance contribution. The manuscript notes that catB3 was truncated in all instances and does not confer chloramphenicol resistance.

This is important. Please make a clearer distinction between loci that are structurally recurrent and loci that remain functionally relevant to phenotype. Otherwise, readers may overinterpret the clinical resistance contribution of all genes within the described blocks.

8. Assembly quality and annotation uncertainty deserve more attention. The analysis combines public long-read assemblies generated over many years and annotates them using several tools. Please clarify whether any assembly quality thresholds were applied and how disagreements between annotation tools were resolved. This is especially important for IS-rich regions, which are prone to assembly fragmentation and annotation inconsistency.

9. The proposed outbreak application is interesting but currently speculative. The manuscript suggests that recognizing recurrent co-localized ARG could provide potential targets for screening during outbreaks. This should be presented more carefully as a potential future application rather than an evidence-based practical recommendation arising from the current study.

Minor comments:

1. Add a study-selection figure. A simple visual workflow for article identification, screening, exclusion, and inclusion would greatly improve readability.

2. Clarify whether repeated or highly related sequences may have inflated the frequency of dominant variants. This is particularly relevant when interpreting the most common structural variants.

3. Consider summarizing the main characteristics of included sequences in the main manuscript rather than relying heavily on supplementary tables. This would improve accessibility for readers.

4. It may help to define key terms early, such as "mosaic", "modular", "block", "variant", etc. to avoid ambiguity.

**********

6. PLOS authors have the option to publish the peer review history of their article (what does this mean?). If published, this will include your full peer review and any attached files.

If you choose “no”, your identity will remain anonymous but your review may still be made public.

**Do you want your identity to be public for this peer review?**  For information about this choice, including consent withdrawal, please see our Privacy Policy.

Reviewer #1: No

**********

[NOTE: If reviewer comments were submitted as an attachment file, they will be attached to this email and accessible via the submission site. Please log into your account, locate the manuscript record, and check for the action link "View Attachments". If this link does not appear, there are no attachment files.]

To ensure your figures meet our technical requirements, please review our figure guidelines: https://journals.plos.org/plosone/s/figures

You may also use PLOS’s free figure tool, NAAS, to help you prepare publication quality figures: https://journals.plos.org/plosone/s/figures#loc-tools-for-figure-preparation.

NAAS will assess whether your figures meet our technical requirements by comparing each figure against our figure specifications.

# Author comment: Author response to Decision Letter 1

**DOI:** 10.1371/journal.pone.0355393.r002

2 Jul 2026

Manuscript: Structural insights into the mosaic and modular organization of fourteen co-occurring resistance genes in multidrug resistant Enterobacteriaceae

Dear reviewer,

We would like to express our thanks for the thorough review and insightful comments on our manuscript. The comments provided were brought forward in a comprehensive and constructive manner which is truly appreciated.

Overall, we agree with the reviewer and have revised the manuscript to improve methodological transparency, clarify analytical decisions and restrain speculative claims.

The text below describes our point-by-point response to the major and minor comments.

Sincerely,

Connor Rossel

Also on behalf of my co-authors

Major comments:

1. Please clarify the methodological nature of the study. The manuscript combines a literature search with downstream comparative genomic analysis, but it is not entirely clear how the study should be classified methodologically. The work has features of a structured review, but it is not reported as a systematic review. This distinction matters because readers need to understand whether the literature identification and screening process was intended to be comprehensive and reproducible. A clearer statement in the Methods would be helpful. A study-selection flow diagram would also improve transparency.

Response:

This is a valid remark, which we have clarified further in the method section (line 83).

We think that the nature of the study can be best described as a structured literature review. The label of systematic literature review would not be appropriate. The core objective of the current study is to collect sequences for secondary genomic analysis rather than to critically appraise and synthesize the findings of the prior studies themselves. Also related to this difference in core objective, the current study does not meet other important criteria considered standard for a systematic review, such as risk-of-bias analyses for the included studies and strict use of indexed biomedical databases.

A study-selection flow diagram has also been added to the results section (Fig 1).

2. The search strategy may enrich for the very structures under investigation. Because the literature search was ARG-centric and required combinations of three to five predefined genes, as well as at least six co-occurring ARG in a long-read assembly, the study design may preferentially retrieve highly relevant examples of these multidrug resistance regions while excluding related but less canonical structures. Please discuss more explicitly how this selection strategy may have biased the observed frequency and apparent recurrence of these ARG blocks.

Response:

This is indeed a possibility. We described this bias more explicitly together with major comment 5 and minor comment 2 in the discussion (line 398-411).

3. The rationale for selecting a threshold of at least six ARG needs to be strengthened. The cutoff is justified by prior observations in the Netherlands, where six genes were linked to a similar MDR phenotype. However, the present manuscript uses this threshold for a global structural analysis. Please explain more clearly why this threshold is appropriate beyond the original local setting. If feasible, a sensitivity analysis using different thresholds would strengthen confidence that the main findings are not an artefact of this choice.

Response:

We chose this specific threshold based on the potential inference of MDR phenotypes against (global cornerstone) antimicrobial categories for the treatment of Enterobacteriaceae infections. This includes non-susceptibility to aminoglycosides by aac(3)-IIe or aac(6')-Ib-cr, to cotrimoxazole by dfrA14 and sul2, to fluoroquinolones by qnrB1 or aac(6')-Ib-cr, and to extended-spectrum β-lactams by blaCTX-M-15. This has been described in the methods (line 91- 96).

As suggested, we applied a post-hoc analysis for the impact of different thresholds on the main findings (line 278-290, S4 Table). Increasing the threshold appears to decrease genomic sequences and structural mosaic variants in a proportionate manner. The mosaic variants lost during the increase of the threshold are those observed in a low frequency. Increasing thresholds (e.g., to ≥8 ARG) does not appear to change the predominant mosaic variants (n > 10) described in the body of the manuscript. Therefore, we do not expect that the main findings are an artefact of the initial cut-off of 6 ARG.

4. Please justify how the nine ARG blocks were defined. The Methods state that the direct environment of each ARG was analyzed to reduce the influence of broader modular rearrangement, and that tandem ARG were grouped into a single resistance element. However, the criteria used to delimit each block are not fully explicit. Were these boundaries defined manually, by fixed rules, or iteratively after inspection? This needs to be described more rigorously, particularly for regions involving IS26, truncated elements, or tandem resistance genes.

Response:

This section has been rewritten in the methods to clarify which considerations were applied in the identification of resistance elements and the demarcation of their boundaries (line 117-146). This includes clarification on regions involving IS26, truncated elements and tandem resistance genes.

5. The dominance of certain taxa and plasmid contexts limits generalizability. Most included sequences were derived from plasmids, and Klebsiella pneumoniae complex was the dominant taxon. This skew should be addressed more directly in the Discussion. It is possible that some of the observed recurrence reflects the repeated recovery of successful plasmid scaffolds or well-sampled lineages rather than broadly distributed architecture across all Enterobacteriaceae.

Response:

This bias is indeed a drawback of the currently used approach. We have stated this concern more explicitly in the limitations of the discussion (line 398-404).

6. Claims regarding transfer should be phrased more cautiously. The conclusion states that preservation of these clinically relevant ARG suggests effective transfer of these resistance elements between distinct isolates. This is plausible, but the study does not directly demonstrate transfer, mobility, or conjugation. At most, the data support recurrent structural association across diverse genomic contexts. I recommend softening the conclusion to reflect that inter-isolate transfer is inferred rather than shown.

Response:

This was indeed our intention. The sentence has been altered to: “the preservation of these clinically relevant resistance elements infers a potential means of transfer between distinct Enterobacteriaceae isolates.”. A follow-up sentence is also added to make this clear: “Further analysis of the plasmid sequences is required to elucidate this means.” (line 429-431)

7. Functional interpretation should distinguish conserved structure from active resistance contribution. The manuscript notes that catB3 was truncated in all instances and does not confer chloramphenicol resistance. This is important. Please make a clearer distinction between loci that are structurally recurrent and loci that remain functionally relevant to phenotype. Otherwise, readers may overinterpret the clinical resistance contribution of all genes within the described blocks.

Response:

Indeed, a few elements have recurrent variants with truncated resistance genes which likely do not confer the associated phenotype anymore. Besides the ΔcatB3 that was truncated in all structures, drfrA14 was truncated in v2791-V7304 (n=8) and v6428 (n=2), and strB was truncated in v1921-v6811 (n=2). (line 249-251 and line 276-277).

We have made mentioned of these variants under the section “Mosaicism of the resistance elements” in their respective paragraphs. We chose to mention this here since these resistance genes were not initially detected with AMRFinderPlus (<60% coverage); it was detected during assessment of the mosaic structure of the resistance elements.

8. Assembly quality and annotation uncertainty deserve more attention. The analysis combines public long-read assemblies generated over many years and annotates them using several tools. Please clarify whether any assembly quality thresholds were applied and how disagreements between annotation tools were resolved. This is especially important for IS-rich regions, which are prone to assembly fragmentation and annotation inconsistency.

Response:

We did not apply any assembly quality threshold and clarified this in the methods section. (line 100).

A clarification for the resolution of discrepant annotation is also added. (line 112-116).

9. The proposed outbreak application is interesting but currently speculative. The manuscript suggests that recognizing recurrent co-localized ARG could provide potential targets for screening during outbreaks. This should be presented more carefully as a potential future application rather than an evidence-based practical recommendation arising from the current study.

Response:

This sentence has been rephrased more cautiously to clearly express the speculative nature. “Hypothetically, these three groups could serve as screening targets during outbreaks of MDR Enterobacteriaceae harboring these co-occurring ARGs; however, further empirical evidence is required to validate their practical utility in epidemiological surveillance.” (line 348-351).

Minor comments:

1. Add a study-selection figure. A simple visual workflow for article identification, screening, exclusion, and inclusion would greatly improve readability.

Response:

We have added a study selection figure in the results section (Fig 1). (line 160)

2. Clarify whether repeated or highly related sequences may have inflated the frequency of dominant variants. This is particularly relevant when interpreting the most common structural variants.

Response:

This has been clarified in the discussion. (line 404-407).

3. Consider summarizing the main characteristics of included sequences in the main manuscript rather than relying heavily on supplementary tables. This would improve accessibility for readers.

Response:

Unfortunately, we cannot determine which specific main characteristics of the sequences are meant here. The characteristics found in supplementary table 2 have been summarized in the main body text of the manuscript. This includes geography, host, taxonomy, and type of sequence (plasmids/chromosome). (line 162-173).

We have adjusted the text slightly in the main text to better indicate in advance what supplementary table 2 contains, should a reader be interested in the sequence specific characteristics.

4. It may help to define key terms early, such as "mosaic", "modular", "block", "variant", etc. to avoid ambiguity.

Response:

We have made some alterations in the introduction of the manuscript to better define these terms early on.

Throughout the manuscript we have also altered the word “block” into “resistance elements”, as both terms were used interchangeably.

Attachment Submitted filename: Response to Reviewers.docx

# Editor report: Decision Letter 1

**DOI:** 10.1371/journal.pone.0355393.r003

**Authors:** Clinton Moodley

22 Jul 2026

Structural insights into the mosaic and modular organization of fourteen co-occurring resistance genes in multidrug resistant Enterobacteriaceae

PONE-D-26-13574R1

Dear Dr. Rossel,

We’re pleased to inform you that your manuscript has been judged scientifically suitable for publication and will be formally accepted for publication once it meets all outstanding technical requirements.

Within one week, you’ll receive an e-mail detailing the required amendments. When these have been addressed, you’ll receive a formal acceptance letter and your manuscript will be scheduled for publication.

An invoice will be generated when your article is formally accepted. Please note, if your institution has a publishing partnership with PLOS and your article meets the relevant criteria, all or part of your publication costs will be covered. Please make sure your user information is up-to-date by logging into Editorial Manager at Editorial Manager® and clicking the ‘Update My Information' link at the top of the page. For questions related to billing, please contact billing support.

If your institution or institutions have a press office, please notify them about your upcoming paper to help maximize its impact. If they’ll be preparing press materials, please inform our press team as soon as possible -- no later than 48 hours after receiving the formal acceptance. Your manuscript will remain under strict press embargo until 2 pm Eastern Time on the date of publication. For more information, please contact onepress@plos.org.

Kind regards,

Clinton Moodley, Ph.D.

Academic Editor

PLOS One

Additional Editor Comments (optional):

The authors have addressed the complex and comprehensive recommended changes from the reviewer. The manuscript is strengthened and significantly improved in readability and meaning throughout.

Reviewers' comments:

# Editor report: Acceptance letter

**DOI:** 10.1371/journal.pone.0355393.r004

**Authors:** Clinton Moodley

PONE-D-26-13574R1

PLOS One

Dear Dr. Rossel,

I'm pleased to inform you that your manuscript has been deemed suitable for publication in PLOS One. Congratulations! Your manuscript is now being handed over to our production team.

At this stage, our production department will prepare your paper for publication. This includes ensuring the following:

* All references, tables, and figures are properly cited

* All relevant supporting information is included in the manuscript submission,

* There are no issues that prevent the paper from being properly typeset

You will receive further instructions from the production team, including instructions on how to review your proof when it is ready. Please keep in mind that we are working through a large volume of accepted articles, so please give us a few days to review your paper and let you know the next and final steps.

Lastly, if your institution or institutions have a press office, please let them know about your upcoming paper now to help maximize its impact. If they'll be preparing press materials, please inform our press team within the next 48 hours. Your manuscript will remain under strict press embargo until 2 pm Eastern Time on the date of publication. For more information, please contact onepress@plos.org.

You will receive an invoice from PLOS for your publication fee after your manuscript has reached the completed accept phase. If you receive an email requesting payment before acceptance or for any other service, this may be a phishing scheme. Learn how to identify phishing emails and protect your accounts at https://explore.plos.org/phishing.

If we can help with anything else, please email us at customercare@plos.org.

Thank you for submitting your work to PLOS ONE and supporting open access.

Kind regards,

PLOS ONE Editorial Office Staff

on behalf of

Dr. Clinton Moodley

Academic Editor

PLOS One
